# Dominant activities of fear engram cells in the dorsal dentate gyrus underlie fear generalization in mice

**DOI:** 10.1371/journal.pbio.3002679

**Published:** 2024-07-12

**Authors:** Kun Cui, Xuetao Qi, Zilong Liu, Weiqi Sun, Peijie Jiao, Chang Liu, Jifu Tong, Xiaoyan Sun, Haojie Sun, Su Fu, Jiaxin Wang, Yawen Zheng, Tianyu Liu, Shuang Cui, Fengyu Liu, Jian Mao, Jie Zheng, You Wan, Ming Yi

**Affiliations:** 1 Neuroscience Research Institute and Department of Neurobiology, School of Basic Medical Sciences, Peking University, Beijing, China; 2 Beijing Life Science Academy, Beijing, China; 3 UCL School of Pharmacy, University College London, London, United Kingdom; 4 Department of Anesthesiology, Peking University People’s Hospital, Beijing, China; 5 Key Laboratory for Neuroscience, Ministry of Education/National Health Commission, Peking University, Beijing, China; 6 Co-innovation Center of Neuroregeneration, Nantong University, Nantong, China; Institute of Experimental Medicine: Kiserleti Orvostudomanyi Kutatointezet, HUNGARY

## Abstract

Over-generalized fear is a maladaptive response to harmless stimuli or situations characteristic of posttraumatic stress disorder (PTSD) and other anxiety disorders. The dorsal dentate gyrus (dDG) contains engram cells that play a crucial role in accurate memory retrieval. However, the coordination mechanism of neuronal subpopulations within the dDG network during fear generalization is not well understood. Here, with the Tet-off system combined with immunostaining and two-photon calcium imaging, we report that dDG fear engram cells labeled in the conditioned context constitutes a significantly higher proportion of dDG neurons activated in a similar context where mice show generalized fear. The activation of these dDG fear engram cells encoding the conditioned context is both sufficient and necessary for inducing fear generalization in the similar context. Activities of mossy cells in the ventral dentate gyrus (vMCs) are significantly suppressed in mice showing fear generalization in a similar context, and activating the vMCs-dDG pathway suppresses generalized but not conditioned fear. Finally, modifying fear memory engrams in the dDG with “safety” signals effectively rescues fear generalization. These findings reveal that the competitive advantage of dDG engram cells underlies fear generalization, which can be rescued by activating the vMCs-dDG pathway or modifying fear memory engrams, and provide novel insights into the dDG network as the neuronal basis of fear generalization.

## Abstract 2

Fear generalization is a maladaptive response to harmless stimuli or situations that is characteristic of posttraumatic stress disorder and other anxiety disorders. This study shows that competition between engram and non-engram cells in the dentate gyrus determines whether fear generalization occurs.

## Introduction

Fear generalization refers to conditioned fear responses that generalize or spread to related stimuli [1]. Since aversive episodes are not always identical, subjects generalize their fear of a past experience to future encounters that bear a sufficient degree of similarity to the original event [2]. Fear generalization can be adaptive, but may also contribute to maladaptive behaviors depending on the context and degree. Over-generalized fear is harmful and a common symptom of anxiety-related disorders, including generalized anxiety disorder, panic disorder, and posttraumatic stress disorder (PTSD) [3–5]. PTSD patients exhibit generalized fear early after experiencing stress, which lasts for a long duration with limited attenuation and substantially harms mental health and quality of life [6,7].

The over-generalized fear response to innocuous cues may result from over-sensitized memory retrieval, implying that dysregulated activity in the hippocampus may contribute to fear generalization. Indeed, PTSD patients are characterized by suppressed hippocampal activity during stimulus discrimination tasks, and the degree of hippocampal inhibition positively correlates with the degree of fear generalization [8]. In the rodent hippocampus, dorsal dentate gyrus (dDG) mediates pattern separation and underlies accurate memory retrieval: mice lacking the subunit of NMDA receptor NR1 in dDG are unable to discriminate between 2 similar contexts and display a fear response in the safe context [9], whereas reducing ABLIM3 in the projection from DG granule cells to CA3 interneurons increases synaptic connectivity and reduces remote contextual fear generalization [10].

However, paradoxical evidence exists regarding the neuronal basis of dDG in memory retrieval. On one hand, activation and inhibition of cFOS-expressing engram cells labeled during fear learning induce and reduce generalized fear behaviors in a novel context with only partial cues, respectively [11,12]. On the other hand, in a novel context with only partial cues, nonselective optogenetic inhibition of the dDG neuronal population increases fear generalization during the memory retrieval test [13]. These results imply that reactivation of dDG engram cells leads to inaccurate memory retrieval, but this process may receive substantial influences from neighboring non-engram neurons [14–16]. However, direct evidence for such interactions between dDG neuronal subpopulations during memory retrieval, as well as their contribution to fear generalization, is lacking. Furthermore, mossy cells in the ventral DG (vMCs) send dense excitatory projections to and modulate neuronal activities of dDG granule cells [17]. The vMCs-dDG pathway mediates cognitive flexibility and novelty detection [17–23], all of which potentially influence the level of fear memory in a novel context with only partial cues, i.e., fear generalization. But the exact role of vMCs in generalized fear is also unclear.

In the present study, we applied function-specific neuronal labeling and two-photon calcium imaging in freely behaving mice to investigate DG neuron network activity in fear generalization, and showed that generalized fear in a novel context resulted from the competitive advantage of fear engram cells in the original context over non-engram cells in the dDG, and that activating the vMC-dDG pathway weakened the competitive advantage of engram cells in the dDG neuronal network and attenuated fear generalization.

## Results

### High reactivation of fear engram cells in dDG parallels fear generalization in a similar context

We first established a contextual fear conditioning (CFC) paradigm and examined whether footshocks in a conditioned context A induced fear generalization in a novel but similar context B (S1 Fig). In the absence of electric shock, the mice displayed limited freezing behavior (below 10%) in both contexts A and B (S1A–S1C Fig), indicating the absence of inherent fear in these contexts. Next, mice were trained with 3 (weak shocks, WS) or 6 (strong shocks, SS) electric shocks in context A to induce conditioned contextual fear. Consistent with previous studies [24], the SS group displayed a similar level of freezing to the WS group in context A (S1D and S1E Fig), but a significantly higher freezing level in context B (S1F and S1G Fig), indicating fear generalization.

**Fig 1:**
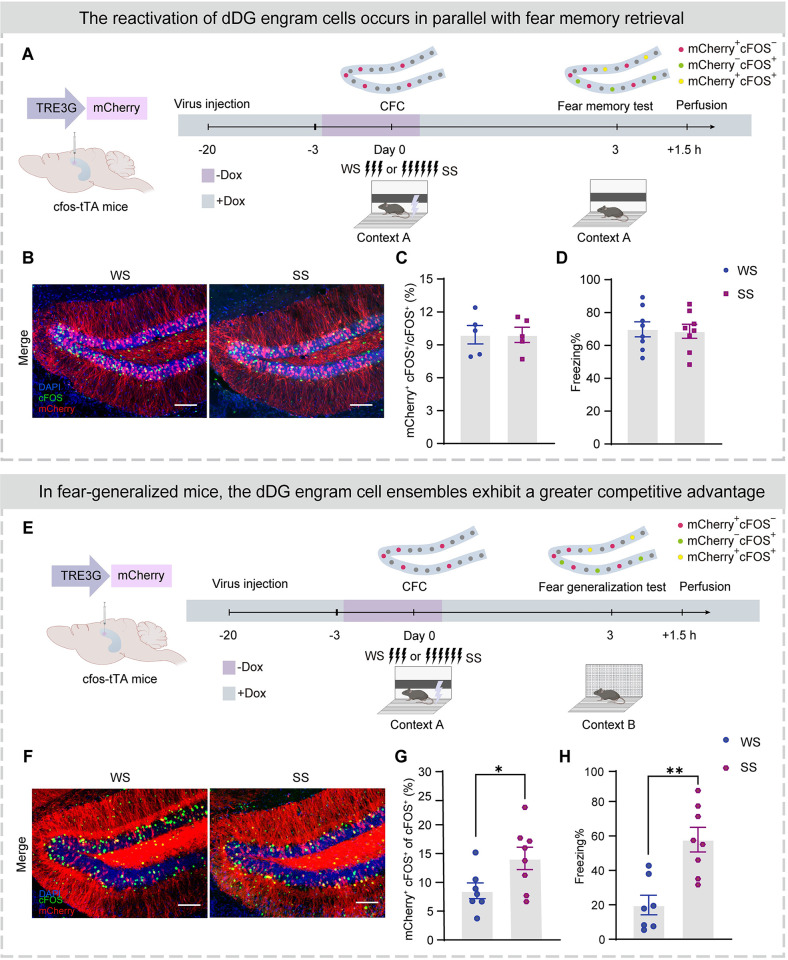
Higher reactivation of fear engram cells in dDG parallels fear generalization in a similar context. (A) Experimental design. After virus expression, mice were taken off Dox for 2.5 days and subjected to CFC training in context A. Three days later, mice were tested in context B and perfused after testing 1.5 h for immunostaining. Top: Schematic to visualize cells active during CFC and fear memory test. The mCherry^+^ cells were labeled cells that were activated during CFC. dDG cells activated during the fear generalization test were visualized with immunostaining for endogenous cFOS 90 min after the test. (B) Representative merged images of mCherry^+^ expressing (red) and cFOS^+^ immunostaining (green) in the dDG. Nuclei in blue (DAPI). Scale bar, 100 μm. (C) Similar proportions of activated engram cells (mCherry^+^ cFOS^+^)/activated cells (cFOS^+^) between SS and WS groups. (D) WS and SS groups exhibited similar levels of freezing during fear memory test. (E) Experimental design. After virus expression, mice were taken off Dox for 2.5 days and subjected to CFC training in context A. Three days later, mice were tested in context B and perfused after testing 1.5 h for immunostaining. Top: Schematic to visualize cells active during CFC and fear generalization test. The mCherry^+^ cells were labeled cells that were activated during CFC. dDG cells activated during the fear generalization test were visualized with immunostaining for endogenous cFOS 90 min after the test. (F) Representative merged images of mCherry^+^ expressing (red) and cFOS^+^ immunostaining (green) in the dDG. Nuclei in blue (DAPI). Scale bar, 100 μm. (G) Higher proportion of activated engram cells (mCherry^+^ cFOS^+^)/activated cells (cFOS^+^) in the SS group than the WS group. (H) The SS group displayed a higher percentage of freezing levels during the fear generalization test. In Fig 1, statistical comparisons were performed using unpaired Student’s *t* test. Data were presented as mean ± SEM. *p < 0.05, **p < 0.01. The underlying data and statistical information in Fig 1 can be found in S1 Data. The mice depicted were created with BioRender.com. CFC, contextual fear conditioning; dDG, dorsal dentate gyrus; SS, strong shock; WS, weak shock.

To examine dDG neuronal activity during CFC training and test, we injected AAV9-TRE3G-mCherry (Table 1) into the dDG of cfos-tTA transgenic mice. The *cfos* promoter controlled the expression of tTA, which combined with TRE3G to express mCherry under the control of a doxycycline diet (Dox, 40 mg/kg). After virus expression, mice were taken off Dox for 2.5 days, and experienced weak or strong shock CFC training in context A, and returned to their homecage with a Dox diet. Based on our pilot experiment, this protocol was sufficient to label dDG neurons activated by CFC training (dDG fear engram cells). Three days later, mice were exposed to context A for a fear memory test (Fig 1A) and cells activated during the test were stained using c-FOS staining. Therefore, the CFC learning-induced engram cells, the cells activated during the fear memory test and the overlapped neuronal subpopulation were labeled with mCherry (mCherry^+^ cell: engram cell), cFOS antibodies (cFOS^+^ cell: cells activated during the test), and mCherry^+^ cFOS^+^ cells (mCherry^+^ cFOS^+^ cell: reactivated engram cell), respectively (Figs 1B, 1C, and S2A–S2D).

**Table 1:** Key resources table.

REAGENT or RESOURCE	SOURCE	IDENTIFIER
**Antibodies**		
Rabbit Anti-cFOS	CST	CAT # 2250S
Mouse Anti-cFOS	Santa Cruz	CAT # sc-166940
Rabbit Anti-Calb2	Abclonal	CAT # A9807
Donkey anti-mouse IgG-Alexa 594	Abcam	CAT # ab150108
Donkey anti-rabbit IgG-Alexa 647	Abcam	CAT # ab150075
**Bacterial and virus strains**		
AAV9-hSyn-GCaMP6f	Taitool Bioscience	Cat# SO224-9-H20
AAV9-hSyn-mCherry	Vigene Technology	N/A
AAV9-hSyn-ChR2-mCherry	Vigene Technology	N/A
AAV9-TRE3G-mCherry	BrainVTA	N/A
AAV9-TRE3G-ChR2-mCherry	BrainVTA	N/A
AAV2/9-TRE3G-NpHR-EYFP	BrainVTA	N/A
AAV-cfos-tTA-TRE3G-mCherry	BrainVTA	N/A
AAV-TRE3G-hM3D(Gq)-mCherry	BrainVTA	N/A
AAV2/retro-hSyn-EGFP	BrainVTA	N/A
AAV2/5-hSyn-DIO-hM3D(Gq)-EGFP	BrainVTA	N/A
AAV2/5-hSyn-DIO-hM4D(Gi)-EGFP	BrainVTA	N/A
**Chemicals**		
Clozapine-N-oxide	Tocris	CAT # 4936/50
DAPI	Cell Signaling Technology	CAT # 4083S
4-Hydroxytamoxifen (4-OHT)	Sigma	CAT # H6278
Experimental models: Organisms/strains		
Mouse: C57BL/6N	Department of Laboratory Animal Sciences, Peking University Health Science Center	JAX 000664
Mouse: cfos-tTA	The Jackson Laboratory	JAX 018306
Mouse: Fos-CreER^T2^	The Jackson Laboratory	JAX 021882
Mouse: Ai9	The Jackson Laboratory	JAX 007905
**Software and algorithms**		
Fiji (ImageJ)	NIH	https://fiji.sc/
GraphPad Prism 9	GraphPad	https://www.graphpad.com/scientificsoftware/prism/
MATLAB	MathWorks	https://www.mathworks.com
Suite2p	HHMI Janelia Research Campus	https://www.suite2p.org/

We observed similar proportions of activated dDG engram neurons during CFC training in WS and SS groups (S2A and S2E Fig). Furthermore, we found similar proportions of reactivated dDG engram neurons among the activated dDG neurons during fear memory test in context A (Fig 1C) between WS and SS groups (approximately 10%), where both groups also exhibited similar levels of freezing (Fig 1D). By sharp contrast, in context B (Figs 1E, 1F, and S2E–S2H), the SS group exhibited a significantly higher proportion of reactivated engram cells within the activated dDG neuronal ensembles than the WS group (Fig 1G). These results indicate that, compared to the WS group, the dDG fear engram cells in the SS group show stronger reactivation within the dDG neuronal network in a similar context, which parallels generalized fear (Fig 1H).

**Fig 2:**
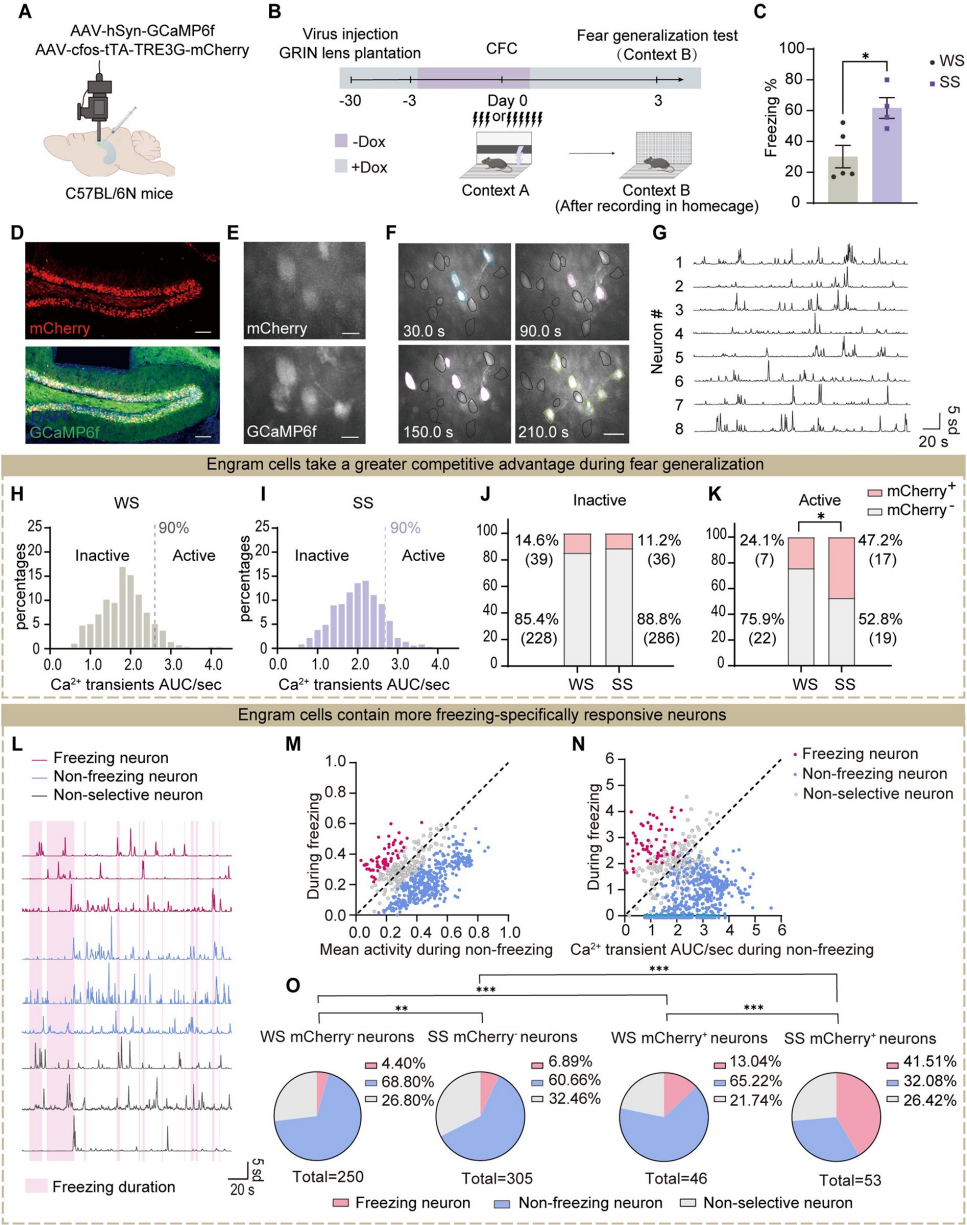
Fear generalization in a similar context accompanies a higher proportion of reactivated fear engram cells. (A) Schematic of virus injection into dDG and a GRIN lens implantation. (B) Experimental design. The dDG calcium activity was recorded during homecage exploration and fear generalization test. (C) The SS group showed higher levels of freezing behavior in context B, indicating fear generalization (unpaired Student’s *t* test). (D) Representative images of mCherry (red) and GCaMP6f (green) expression in the dDG. Nuclei in blue (DAPI). Scale bar, 100 μm. (E) Examples of mCherry expression (top) and GCaMP6f signal (bottom) in vivo images. Scale bar, 20 μm. (F) Examples of GCaMP6f signals in vivo fluorescence images. Neurons with high calcium activity at a specific moment were indicated by different colors. Scale bar, 50 μm. (G) Representative calcium traces. (H) and (I) Histograms of Ca^2+^ transients AUC/sec distribution for individual dDG neurons in the WS (H) and SS (I) groups during fear generalization in context B. (J) During the fear generalization test, the proportions of inactive engram cells (inactive mCherry^+^) and non-engram cells (inactive mCherry^-^) among all inactive cells, respectively. The proportion was similar between WS and SS groups (Chi-squared test). (K) During the fear generalization test, the proportions of active engram cells (active mCherry^+^) and non-engram cells (active mCherry^-^) among all active cells, respectively. Compared to the WS group, the SS group showed a higher proportion of active engram cells (Chi-squared test). (L) Representative Ca^2+^ transients of freezing neurons (dark pink), non-freezing neurons (aquamarine), and nonselective neurons (gray) in the dDG. In the background, pink and white referred to freezing and non-freezing duration, respectively. (M) Mean activity of calcium transient during non-freezing vs. freezing state. (N) Calcium transient AUC/second during non-freezing vs. freezing state. (O) The proportion of 3 types of neurons in the mCherry^-^ and mCherry^+^ neuronal populations of the WS and SS groups. Compared to other neuronal ensembles, the mCherry^+^ neuron ensemble in the SS group contained a higher proportion of freezing neurons (Chi-squared test). In Fig 2, statistical comparisons were performed using unpaired Student’s *t* test or Chi squared test. *p < 0.05, **p < 0.01, ***p < 0.001. The underlying data and statistical information in Fig 2 can be found in S1 Data. The mice depicted were created with BioRender.com. dDG, dorsal dentate gyrus; SS, strong shock; WS, weak shock.

To further confirm these findings, we performed in vivo two-photon recording in freely behaving mice to directly monitor dDG activity dynamics (Fig 2). We injected evenly mixed AAV-cfos-tTA-TRE3G-mCherry and AAV-hSyn-GCaMP6f virus into the dDG of C57BL/6N mice to visualize the activity of local neurons, including the labeled fear engram cells and neighboring other non-engram cells (Fig 2A). This strategy allowed the engram cells to express mCherry and GCaMP6f (mCherry^+^ GCaMP6f^+^), while the non-engram cells only expressed GCaMP6f (mCherry^-^ GCaMP6f^+^). The activity of individual dDG neurons was recorded during staying in the homecage and fear generalization test (Fig 2B–2G). We conducted an analysis of the recorded calcium activity of dDG neurons and generated frequency distribution histograms (Figs 2H, 2I, S3A, and S3B). The Ca^2+^ transients AUC/sec of dDG neurons from each group was sorted in ascending order. The lower 90% were considered inactive neurons, while the top 10% represented active neurons. We analyzed the proportion of active neurons among engram cells (mCherry^+^) and non-engram cells (mCherry^-^) in mice during exploration in the homecage and fear generalization test, respectively, and found a similar proportion of engram versus non-engram cells among the active and inactive populations during homecage exploration (S3C and S3D Fig). By contrast, the active cells during the fear generalization test contained significantly higher proportions of fear engram cells in the SS than in the WS group (Fig 2K). No proportion difference was observed in inactive cells (Fig 2J). Additionally, in terms of behavior, during the fear generalization test, the SS group displayed higher freezing levels, indicating fear generalization (Fig 2C).

Together, these data indicate higher reactivation of fear engram cells within the dDG neuronal network in a similar context, which parallels fear generalization behaviors.

In addition, we conducted an exploration of the characteristics of engram cells in fear-generalized mice: we analyzed whether the engram cells contained neurons exhibiting freezing-specific activity. We found 3 types of responsive selectivity of dDG neurons (Fig 2L–2N): neurons activated during the freezing state (freezing neurons), neurons activated during the non-freezing state (non-freezing neurons), and nonselectively responsive neurons (nonselective neurons). Notably, as expected, the SS group had more freezing neurons among engram cells than the WS group (Fig 2O). These results indicate that the engram cells in fear-generalized mice contain a greater proportion of neurons that exhibit freezing-specific responses.

### dDG fear engram cell activities are both sufficient and necessary for fear generalization in a similar context

**Fig 3:**
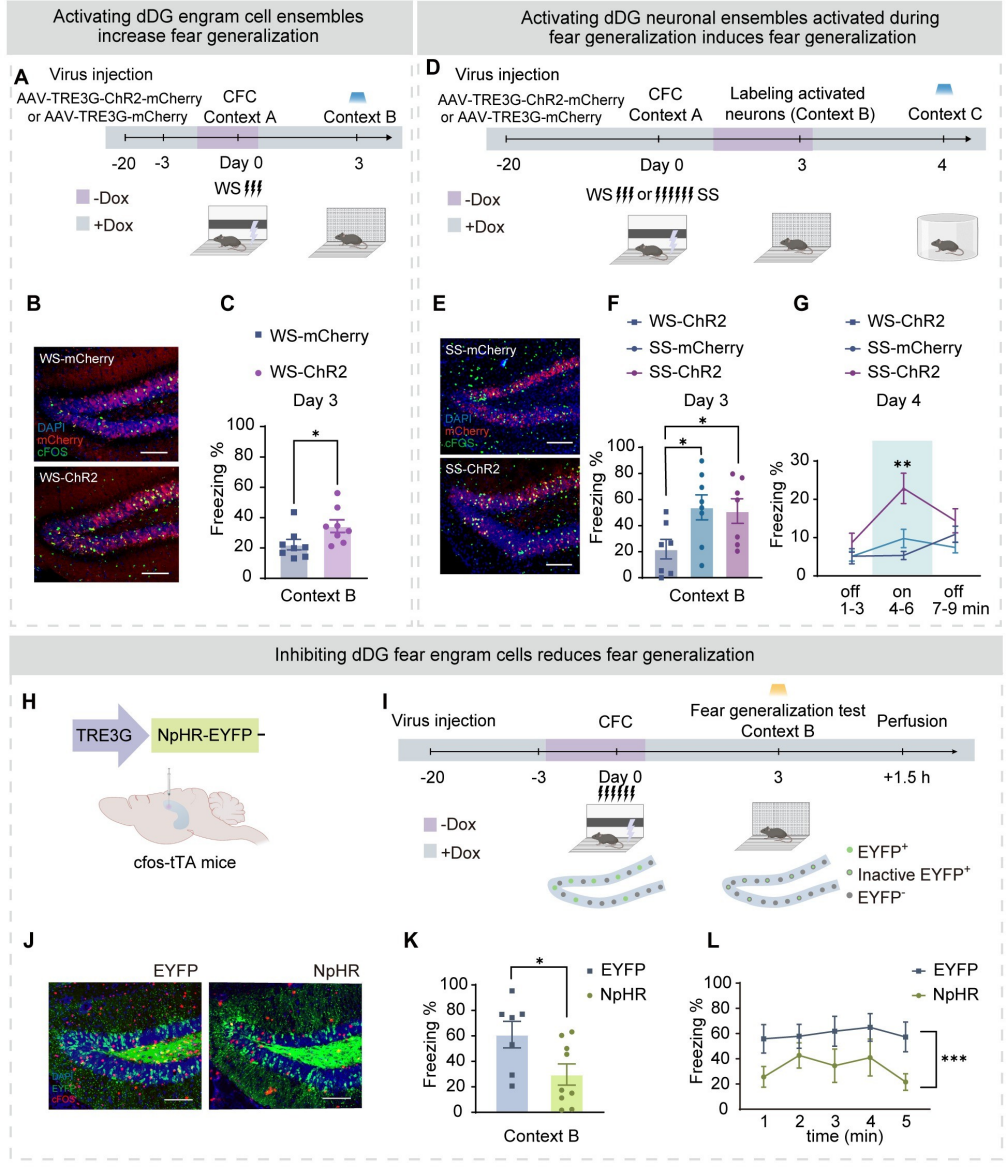
Activated dDG fear engram cells are both sufficient and necessary for fear generalization. (A) Experimental design. (B) Representative images of mCherry^+^ expressing (red) and cFOS^+^ immunostaining (green) in the dDG. Top: WS-mCherry group. Bottom: SS-ChR2 group. Scale bar, 100 μm. (C) Compared with WS-mCherry group, WS-ChR2 group displayed a higher percentage of freezing levels during the fear generalization test (unpaired Student’s *t* test). (D) Experimental design. (E) Representative images of mCherry^+^ expressing (red) and cFOS^+^ immunostaining (green) in the dDG. Left: SS-mCherry group. Right: SS-ChR2 group. Scale bar, 100 μm. (F) The WS-ChR2 group showed a low level of freezing, while the SS-mCherry group and SS-ChR2 group showed a high level of freezing in context B (one-way ANOVA with Bonferroni’s post hoc test). (G) During laser presentation, the SS-ChR2 group showed a high level of freezing in context C (two-way ANOVA with Bonferroni’s post hoc test). (H) Optogenetic design. The dDG of cfos-tTA mice was injected with AAV9-TRE3G-EYFP (EYFP group) or AAV9-TRE3G-NpHR-EYFP (NpHR group) virus. The optic fiber was embedded above the dDG. (I) Experimental design. (J) Representative images of EYFP^+^ expression (green) and cFOS^+^ immunostaining (red) in the dDG. Left: EYFP group. Right: NPHR group. Scale bar, 100 μm. (K) The NPHR group displayed a lower percentage of freezing levels during the memory recall test (unpaired Student’s *t* test). (L) The NPHR group displayed a lower percentage of freezing levels during the fear generalization test from 1 min to 5 min (two-way ANOVA with Bonferroni’s post hoc test). In Fig 3, data were presented as mean ± SEM. *p < 0.05, **p < 0.01, ***p < 0.001. The underlying data and statistical information in Fig 3 can be found in S1 Data. The mice depicted were created with BioRender.com. dDG, dorsal dentate gyrus; SS, strong shock; WS, weak shock.

The above findings suggest that the activity of dDG fear engram cells might mediate fear generalization in a novel context. To causally validate this hypothesis, we conducted CFC with weak shocks (WS) in mice, during which we labeled the activated dDG neurons using the tet-off system (Fig 3A and 3B). After 3 days, we placed the mice in context B for the fear generalization test, during which we activated the labeled dDG engram cells (WS-ChR2 group) and found that compared to the WS-mCherry (control) group, the WS-ChR2 group exhibited higher freezing levels during the fear generalization test (Fig 3C). This suggests that activating dDG engram cells induces fear generalization-like behavior in a novel context. We further investigated whether activating the dDG neuronal ensembles during fear generalization was sufficient to induce fear in a distinct novel context. Therefore, we conducted CFC with either strong (SS) or weak (WS) shocks and labeled the activated dDG neuronal clusters during the fear generalization test. Subsequently, we assessed the effect of activating this labeled dDG neuronal ensembles in a novel context C, which differed substantially from both contexts A and B (Fig 3D–3F). We found that compared to the WS-ChR2 group and the SS-mCherry group, the SS-ChR2 group exhibited higher freezing levels (Fig 3G), indicating that activating the dDG neurons labeled during fear generalization was sufficient to induce fear in a safe context (Fig 3F and 3G).

We next sought to examine whether dDG engram cell activities were necessary for generalized fear, by inhibiting fear engram cells in the similar context (Fig 3H–3L). We injected AAV-TRE3G-NpHR-EYFP to label the dDG engram cells activated during CFC and inhibited the labeled neurons during either re-exposure in context A or the fear generalization test (Fig 3K and 3L). As expected, freezing behavior in both contexts A and B was significantly reduced (Figs 3H–3L and S4).

Together, these findings suggest that dDG fear engram cell activities are both sufficient and necessary for fear generalization in a novel context.

### Suppressing the competitive advantage of dDG engram cells reduces fear generalization

Due to lateral inhibition, dDG neurons exhibit sparse activity and compete with each other [25–29], resulting in predominant activity of a small number of neurons in the whole dDG neuronal network [15,30,31]. Based on the findings above, we hypothesized that competition between dDG fear engram and non-engram subpopulations might underlie the occurrence of fear generalization in a novel context. To test this hypothesis, we employed 2 approaches to artificially reduce the competitive advantage of fear engram cells within the dDG network.

**Fig 4:**
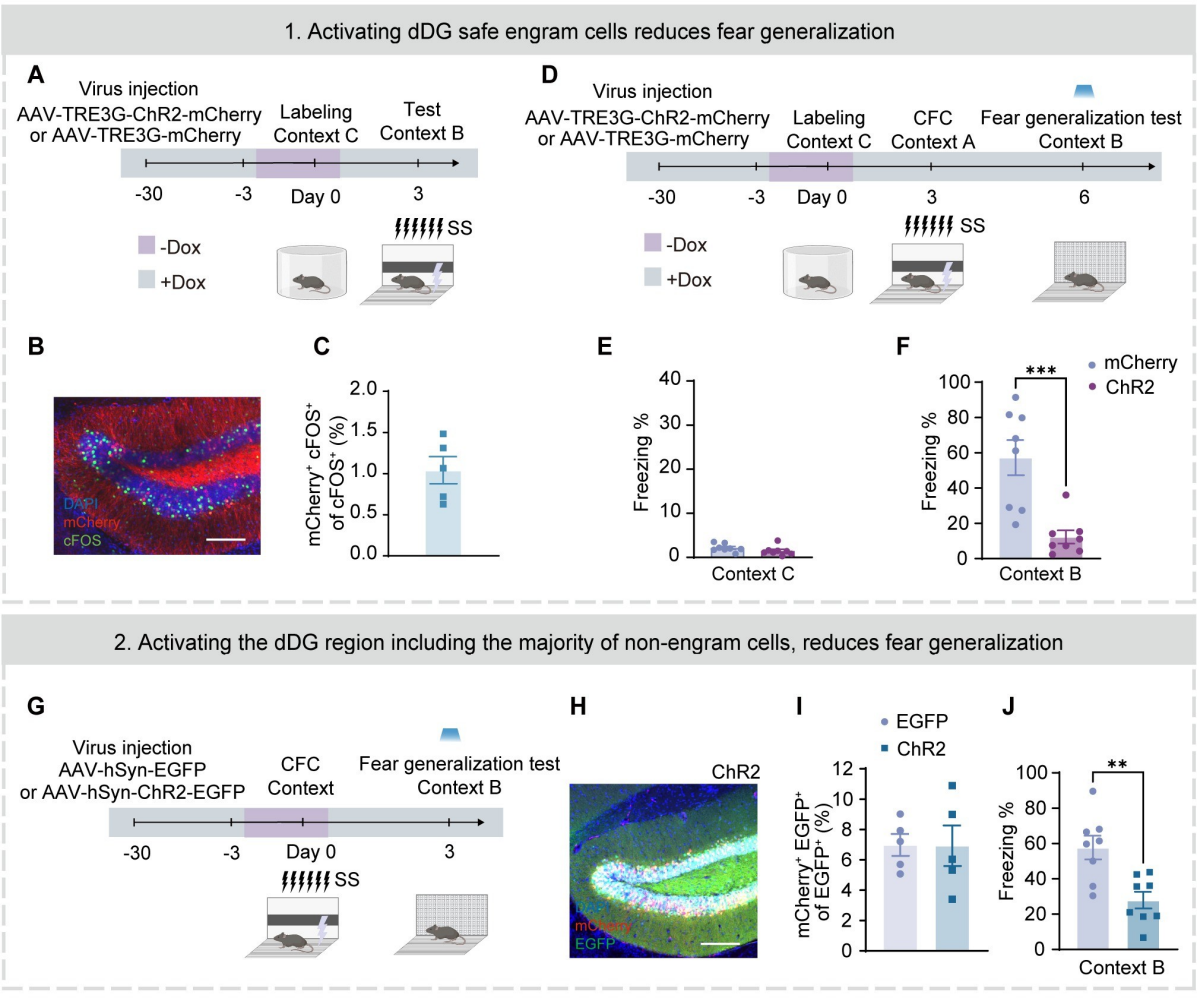
Suppressing the competitive advantage of engram cells in the dDG neuronal network attenuates fear generalization. (A) Experimental design. (B) Representative images of mCherry^+^ expressing (red) and cFOS^+^ immunostaining (green) in the dDG. Nuclei in blue (DAPI). Scale bar, 100 μm. (C) The neurons that are activated in Context C (mCherry^+^) and the neurons that are activated in context B (cFOS^+^) have only a limited overlap (mCherry^+^ cFOS^+^/cFOS^+^). (D) Experimental design. (E) Both mCherry and ChR2 groups showed limited freezing behaviors in context C (unpaired Student’s *t* test). (F) The ChR2 group displayed a lower percentage of freezing levels during the fear generalization test (unpaired Student’s *t* test). (G) Experimental design. (H) Representative images of mCherry^+^ (red) and EGFP^+^ (green) expressing in the dDG. Nuclei in blue (DAPI). Scale bar, 100 μm. (I) Similar proportions of activated engram cells (mCherry^+^ EGFP^+^)/neurons (EGFP^+^) between EGFP and ChR2 groups. (J) The ChR2 group displayed a lower percentage of freezing levels during the fear generalization test (unpaired Student’s *t* test). Data were presented as mean ± SEM. **p < 0.01, ***p < 0.001. The underlying data and statistical information in Fig 4 can be found in S1 Data. The mice depicted were created with BioRender.com. dDG, dorsal dentate gyrus.

We first placed the mice into context C, which differed from both contexts A and B, to label “safety” neurons. Three days later, the mice were placed into context A for CFC training with SS (Fig 4A and 4D). In this safe context, mice showed a low level of freezing behavior (Fig 4E). Considering the sparse firing feature of dDG neurons [32–36], the labeled “safety” neurons would have limited overlap with those activated in context A and represent an independent subpopulation from the fear engram cells, as confirmed by our co-staining experiment (Fig 4A–4C). During the fear generalization test, we activated these labeled dDG neurons in context B and observed significantly reduced freezing behaviors (Fig 4F).

Next, we nonselectively activated the general dDG neuronal population (Fig 4G), where fear engram cells (mCherry^+^) constituted only a minority (Fig 4H and 4I), during fear generalization test, and also observed reduced freezing behaviors during the test (Fig 4J).

Together, these results suggest that the competitive advantage of engram cells in the dDG neuronal network underlies fear generalization.

### Suppressed vMCs activity accompanies with fear generalization

**Fig 5:**
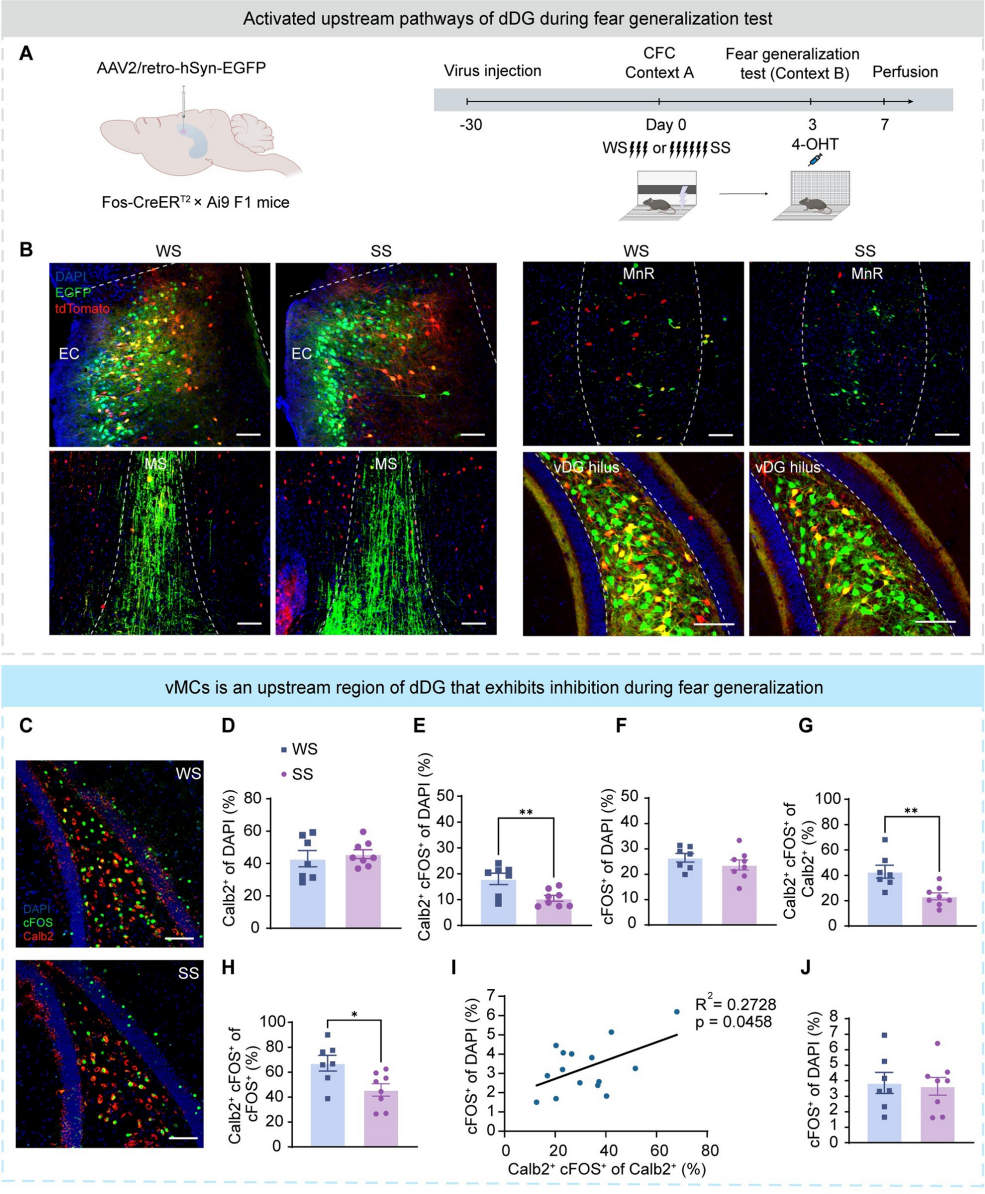
Suppressed vMCs activity parallels fear generalization. (A) Experimental design. Left: Administration of 4-OHT to Fos-CreER^T2^ × Ai9 F1 mice activates permanent expression of a reporter in neurons activated around the time of the injection. To trace dDG afferent projections, the AAV2/retro-hSyn-EGFP virus was injected into the dDG of F1 mice. Right: After virus expression, mice were subjected to CFC training under WS or SS in context A. After 3 days, mice were intraperitoneally injected with 4-OHT and tested in context B. Four days later, mice were perfused. (B) Representative images of tdTomato^+^ (red) cells labeled in context B and dDG afferent projecting neuron expressed EGFP (green). Nuclei were stained with DAPI (blue). The image on the left was from the WS group, and the image on the right was from the SS group. From top to bottom, the brain regions were the entorhinal cortex, MS, MnR, and vDG. Scale bar, 100 μm. (C) Representative image of Calb2^+^ (red) and cFOS^+^ (green) immunostaining in the vDG. Nuclei were stained with DAPI (blue). Scale bar, 100 μm. (D) The proportion of vMCs (Calb2^+^) to all cells (DAPI^+^). A similar percentage of vMCs among all cells in the vDG hilus between the WS and SS groups. (E) The proportion of activated vMCs (Calb2^+^ cFOS^+^) to all cells (DAPI^+^). A lower percentage of activated vMCs among all cells in the hilus of the SS group. (F) The proportion of activated cells (cFOS^+^) to all cells (DAPI^+^). A similar percentage of activated cells among all cells in the vDG hilus between the WS and SS groups. (G) The proportion of activated vMCs (Calb2^+^ cFOS^+^) to all vMCs (Calb2^+^). A lower percentage of activated vMCs among vMCs in the vDG hilus of the SS group. (H) The proportion of activated vMCs (Calb2^+^ cFOS^+^) to all activated cells (cFOS^+^). A lower percentage of activated vMCs among all activated cells in the vDG hilus of the SS group. (I) Pearson correlation test was used to assess the correlation between the percentage of activated vMCs among vMCs (X-axis: Calb2^+^ cFOS^+^ of Calb2^+^ %) and activated dDG cells (Y-axis: cFOS^+^/DAPI %) among DAPI^+^ cells in the dDG. R = Pearson correlation coefficient. (J) The proportion of activated vDG granular cells (cFOS^+^ cells among DAPI^+^ cells in the vDG granular cell layer). A similar percentage of activated vDG granular cells between the WS and SS groups. In Fig 5D–5H and 5J, statistical comparisons were performed using unpaired *t* tests. Data were presented as mean ± SEM. *p < 0.05, **p < 0.01, ***p < 0.001. The underlying data and statistical information in Fig 5 can be found in S1 Data. The mice depicted were created with BioRender.com. CFC, contextual fear conditioning; dDG, dorsal dentate gyrus; MnR, median raphe; MS, medial septum; SS, strong shock; vDG, ventral dentate gyrus; WS, weak shock.

To explore the circuitry basis of fear generalization, we injected the AAV-retro-EGFP virus into the dDG of hybrid F1 generation by crossing male Fos-CreER with female Ai9 (Fig 5A). This system enabled temporally controlled labeling of neurons activated brain-wide during the fear generalization test. Activated afferent neurons of dDG were identified by the expression of EGFP and tdTomato fluorophores, including entorhinal cortex, medial septum (MS), median raphe (MnR), and ventral dentate gyrus (vDG) (Fig 5B).

Among them, the vDG had dense projections to the dDG and was activated in both SS and WS groups during the fear generalization test (Fig 5B). Previous research has shown that mossy cells in vDG (vMCs) are glutamatergic neurons and have dense excitatory projections to almost all dDG granule cells [17]. We counted the neurons stained for cFOS and Calb2, a vMCs-specific marker, (Fig 5C–5H), and found that the activation of vMCs was significantly reduced in the SS group (Fig 5E). By contrast, the activation of vDG granule cells was not associated with fear generalization (Fig 5J). We also failed to observe alterations in cFOS expression levels in the vMCs of mice re-exposed in the fear memory test (context A) (S5 Fig), suggesting that vMC suppression was specific to generalized fear. We further found that after the fear generalization test, the number of activated vMCs positively correlated with the number of activated dDG granule cells (Fig 5I). Since the overall neuronal activity in the dDG was suppressed in the SS group during the fear generalization test (Figs 5I and S1F), these results implied that the inhibition of vMCs might be a circuitry basis of the dDG neuronal network activity pattern associated with fear generalization.

### Activating vMCs-dDG pathway decreases fear generalization

**Fig 6:**
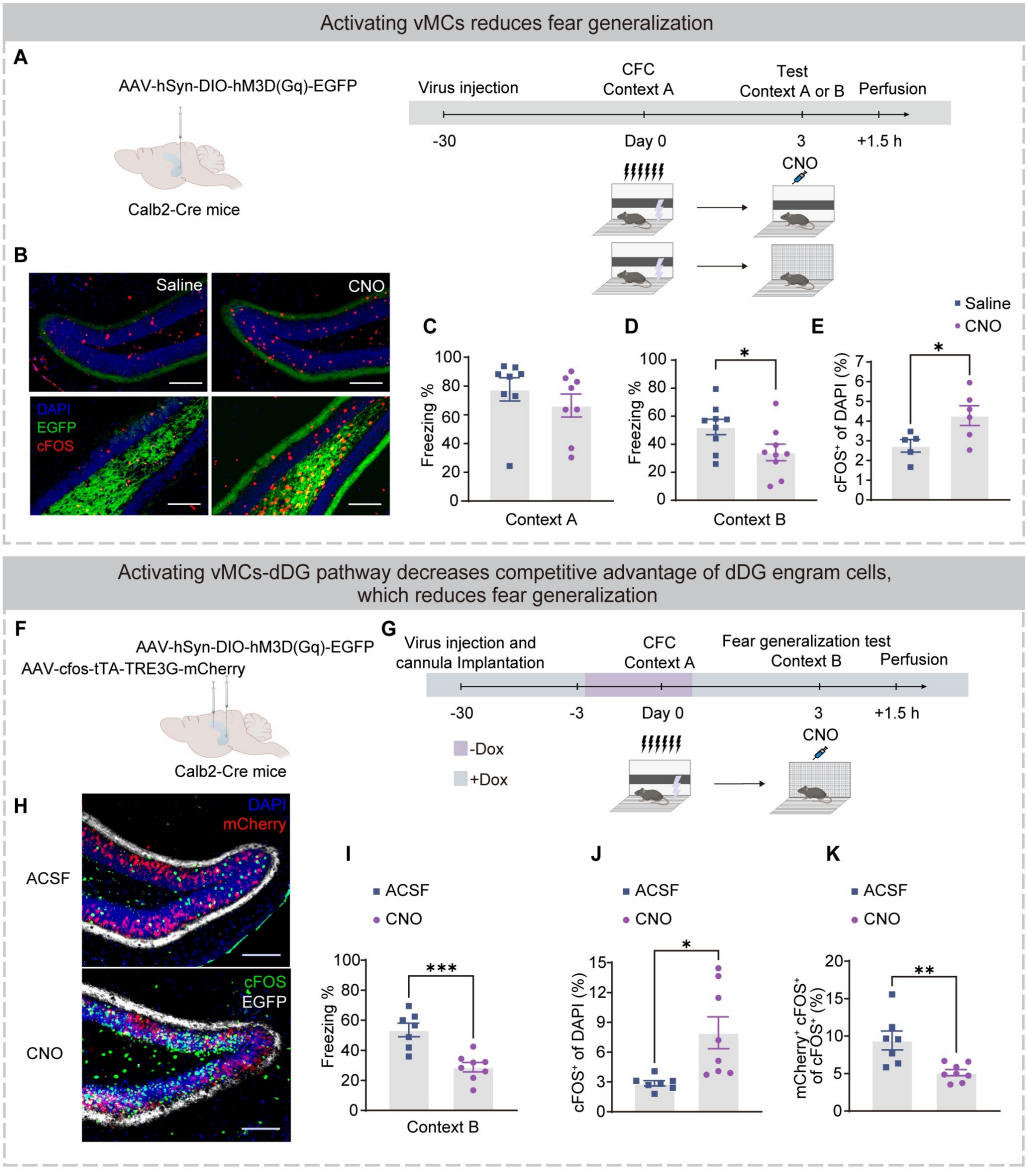
Activation of vMC-dDG pathway decreases the competitive advantage of fear engram cells in the dDG neuronal network and attenuates fear generalization. (A) Experimental design. Left: The vDG of Calb2-Cre mice were injected with AAV-hSyn-DIO-hM3D(Gq)-EGFP virus. Right: After virus expression, mice were subjected to CFC training under SS in context A. After 3 days, mice were intraperitoneally injected with saline (Saline group) or CNO (CNO group), and 40 min later, mice were placed in context A (fear memory test) or context B (fear generalization test), and 1.5 h after testing, mice were perfused for immunostaining. (B) Representative images of EGFP^+^ expressing (green) and cFOS^+^ immunostaining (red) in the dDG (top) and vDG (bottom). Left: Saline group. Right: CNO group. EGFP^+^ fibers indicated axons in the dDG originating from the vMCs cells. Nuclei in blue (DAPI). Scale bar, 100 μm. (C) The Saline and CNO groups showed a similar freezing level in context A during the fear memory test. (D) The CNO group displayed a lower level of freezing during the fear generalization test in context B. (E) The percentage of cFOS^+^ cells. The dDG granular cells of CNO group displayed a significantly greater percentage of activated dDG cells (cFOS^+^) than that of the saline group. (F and G) Experimental design. (F) The vDG of Calb2-Cre mice was injected with AAV-hSyn-DIO-hM3D(Gq)-EGFP virus and the dDG were injected with AAV-cfos-tTA-TRE3G-mCherry virus. (G) After virus expression, mice were subjected to CFC training under SS in context A. After 3 days, mice were injected with ACSF (ACSF group) or CNO (CNO group) intracranially with the injection cannula; 15 min later, mice were placed in context B to test, and 1.5 h after testing, mice were perfused for immunostaining. (H) Representative images of EGFP^+^ expression (white), mCherry^+^ expression (red), and cFOS^+^ immunostaining (green) in the dDG. Left: Saline group. Right: CNO group. Nuclei were stained with DAPI (blue). Scale bar, 100 μm. (I) The CNO group displayed a lower level of freezing during the fear generalization test in context B, indicating fear generalization. (J) The percentage of cFOS^+^ cells. The dDG granular cells of CNO group displayed a significantly greater percentage of activated dDG cells (cFOS^+^) than that of the ACSF group. (K) Activated engram cells (mCherry^+^ cFOS^+^)/activated cells (cFOS^+^). Compared to the ACSF group, the proportion of activated engram cells among all activated cells in the CNO group is lower. Statistical comparisons were performed using unpaired *t* tests. Data were presented as mean ± SEM. *p < 0.05, **p < 0.01, ***p < 0.001. The underlying data and statistical information in Fig 6 can be found in S1 Data. The mice depicted were created with BioRender.com. ACSF, artificial cerebrospinal fluid; CFC, contextual fear conditioning; dDG, dorsal dentate gyrus; SS, strong shock.

We next verified whether the vMCs-dDG pathway causally mediated fear generalization (Figs 6 and S6–S8). The vMCs have limited output projections, with dDG granule cells being the only nonlocal targets reported [17,37]. In addition, vMCs have a significant excitatory effect on the activity of dDG granule cells but mild inhibition on vDG granule cells [17,38]. Therefore, we injected AAV-hSyn-DIO-hM3D(Gq)-EGFP virus in the vDG of Calb2-Cre mice. Saline or CNO was injected intraperitoneally before the fear generalization test (in context B) or fear memory test (in context A) (Fig 6A). CNO effectively activated vMCs (Fig 6B and 6E) and reduced freezing behavior in context B (Fig 6D) but not in context A (Fig 6C). However, injection of AAV-hSyn-DIO-hM4D(Gi)-mCherry virus and inhibition of vMCs with CNO did not affect freezing behaviors during the fear generalization test (S6A–S6E Fig), suggesting that inhibition of vMCs was necessary but not sufficient for fear generalization. These results suggest that activation of the vMCs pathway reduces fear generalization in mice with high levels of fear generalization, but inhibition of the vMCs pathway is not sufficient to induce fear generalization in naive mice.

To further understand the underlying neuronal mechanisms, we analyzed the effect of vMCs-dDG activation on dDG neurons during the fear generalization test. To achieve this, we injected AAV-hSyn-DIO-hM3D(Gq)-EGFP virus in vMCs and AAV-cfos-tTA-TRE3G-mCherry virus in dDG to label engram cells (Fig 6F–6H). The axonal terminals of vMCs projecting to dDG were activated by cannula injection of CNO during the fear generalization test (Fig 6H). The CNO group exhibited higher expression of cFOS in the dDG (Fig 6J), suggesting that activating vMCs-dDG pathway led to enhanced dDG activities. Moreover, compared to the ACSF group, the CNO group exhibited a lower proportion of reactivated engram cells (mCherry^+^ cFOS^+^) among the activated cells (cFOS^+^) (Fig 6K). We also analyzed the proportions of activated engram cells (mCherry^+^ cFOS^+^) and activated non-engram cells (mCherry^-^ cFOS^+^). Notably, activation of the vMCs-dDG pathway preferentially activates non-engram cells in the dDG (S8E Fig), rather than engram cells (S8D Fig). In terms of behavior, compared to the ACSF group, CNO group showed lower freezing levels during fear generalization test (Fig 6I). The direct effect of CNO on dDG activity or fear generalization was excluded by our control experiment utilizing AAV viruses devoid of DREADD (S6F–S6I and S7 Figs). These results suggest that activation of the vMCs-dDG pathway preferentially enhances activity of the non-engram cells subpopulation, leading to a reduction in the relative activity advantage of engram cells in the dDG neuronal network and reduced generalized fear in a similar context (Figs 6I–6K and S8E).

### Modifying fear memory engrams in dDG effectively rescues fear generalization

**Fig 7:**
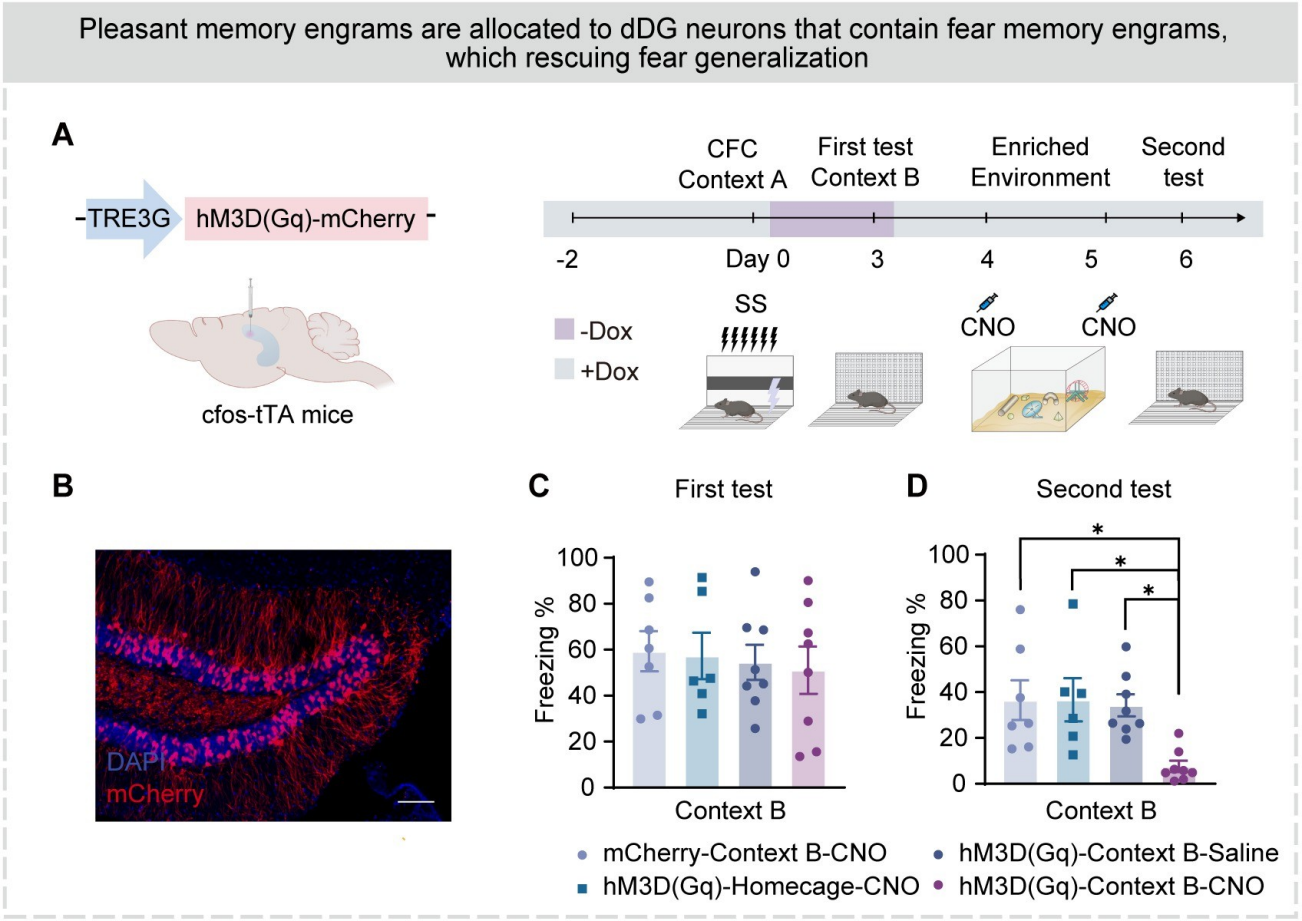
Modifying fear memory engrams in dDG effectively rescues fear generalization. (A) Experimental design. See the METHOD DETAILS section for details. (B) Representative images of mCherry^+^ expressing (red) in the dDG of hM3D(Gq)-Context B-CNO group. Nuclei in blue (DAPI). Scale bar, 100 μm. (C) In the first fear generalization test, the freezing level of the 4 groups of mice was high (one-way ANOVA with Bonferroni’s post hoc test). (D) In the second fear generalization test, mice in the hM3D(Gq)-Context B-CNO group showed decreased freezing levels compared to other groups (one-way ANOVA with Bonferroni’s post hoc test). Data were presented as mean ± SEM. *p < 0.05, **p < 0.01, ***p < 0.001. The underlying data and statistical information in Fig 7 can be found in S1 Data. The mice depicted were created with BioRender.com. dDG, dorsal dentate gyrus.

Activating dDG fear engram cells during a safe event leads to the allocation of safe memory engram, resulting in a switch in emotional valence from negative to positive [39]. In enriched environments, mice exhibit positive emotions and higher levels of synaptic plasticity [40], which facilitates the allocation of new safe memory engrams [39]. To investigate whether modifying the fear memory engram in the dDG could attenuate generalized fear, we first labeled the dDG neurons activated during fear generalization in context B, and chemogenetically activated these dDG cells (Fig 7A–7C) in a safe enriched environment. We observed a significantly reduced freezing level in the following exposure in context B (Fig 7D), indicating that long-term activation of dDG neurons mediating fear generalization in an enriched environment persistently alleviated fear generalization.

## Discussion

### The competitive advantage of fear engram cells in dDG neuronal network underlies fear generalization

**Fig 8:**
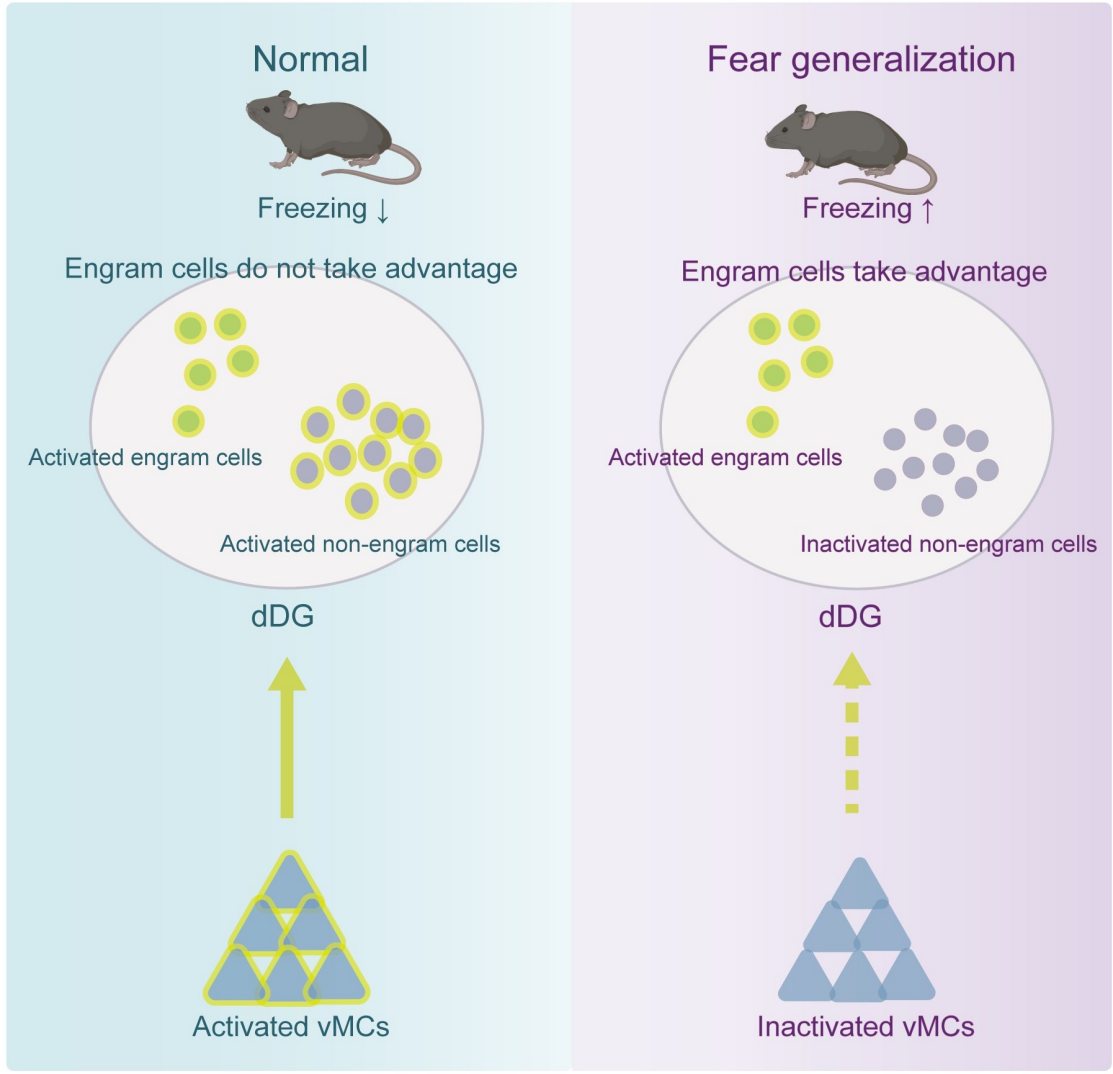
Dentate gyrus mechanism of fear generalization. Right: When the activities of dDG engram cells have a competitive advantage in the dDG neuronal network, fear generalization occurs. Meanwhile, vMCs exhibit suppressed activities. Left: When engram cells do not take great advantage in the dDG network, fear generalization does not occur. In addition, activation of the vMCs-dDG pathway produced biasedly activation of non-engram cells in the dDG, reducing the competitive advantage of engram cells in the dDG neural network and attenuating fear generalization. The underlying data and statistical information in Fig 8 can be found in S1 Data. The mice depicted were created with BioRender.com. dDG, dorsal dentate gyrus; vMC, ventral dentate gyrus.

Mice with fear generalization in a similar context exhibit a higher proportion of active fear engram cells among the activated dDG neurons compared to those without (Figs 1G and 2K). Moreover, it is observed that the heightened activity of dDG engram cells within the dDG neural network confers a competitive advantage, thereby contributing to fear generalization (Fig 3A–3G). Conversely, when the competitive advantage of engram cells in the dDG neuronal network is artificially reduced, it results in decreased fear generalization (Figs 3H–3L and 4). If fear generalization is regarded as a more effective and sensitive form of memory retrieval, these results suggest that SS may lead to the formation of stronger engrams, triggered by various subtle contextual cues from the conditioned context (context A). The enhanced reactivation of engram cells in Fig 1G is attributed to the more specific responsiveness of engram cells than non-engram cells in the “novel but similar context,” thus facilitating the retrieval of fear memories more effectively. This phenomenon reflects that the engram cells induced by stronger stimuli (i.e., SS) are more efficient in prioritizing specific activation in this novel context. In summary, these findings suggest that the competitive advantage of engram cells within the dDG neuronal network is a key factor underlying fear generalization (Fig 8).

Previous studies have shown that abnormal activity of dDG is involved in fear generalization [41], and that the activity of dDG engram cells is related to the occurrence of fear generalization [12]. However, due to technical limitations, these studies did not trace the activity dynamics of the dDG neuronal network composed of both engram and non-engram cells, but only emphasized the role of the dDG engram cell population or the entire dDG region in fear generalization [11–13], leading to seemingly contradictory conclusions [13]. Our results explain these paradoxical findings by showing that the competition between neuronal populations in the dDG network determines whether fear generalization occurs. When non-engram cells dominate in the dDG network, fear generalization does not occur, while engram cells dominating leads to fear generalization. This phenomenon may be due to the characterized by an attractor-like “winner-takes-all” feature, where dDG neurons’ sparse activity and mutual competition [9,27–29,42–44]. As a result, active fear engram cells in the dDG dominate the downstream output region CA3 and causes generalized fear. Previous studies have shown that the competition between different neuronal ensembles in the amygdala determines whether fear memories are expressed [45,46]. Unlike the amygdala, the dDG plays an important role when individuals encounter ambiguous cues [13,30]. The competition between dDG neurons determines the accuracy of memory retrieval, specifically whether fear generalization occurs.

In addition, activation of “safe” engram cells reduces fear generalization (Fig 4D–4F), where the “safe” context may have to be a novel context, where mice have a stronger memory encoding in dDG during labeling. Conversely, safe engram cells labeling in a familiar safe contexts may be less able to compete with fear engram cells [47].

This study mainly focused on the role of dDG and its engram cells in fear generalization, but did not pay attention to vDG engram cells. Previous studies have shown that acute or chronic activation of vDG engram cells does not seem to reduce contextual fear memory, but instead mediates aversion, similar to the basolateral amygdala (BLA) [48].

### vMCs-dDG pathway weakens the competitive advantage of fear engram cells in the dDG neuronal network and attenuates fear generalization

Due to their sensitivity to environmental information and tendency to prioritize new information, vMCs are considered the “sentinel” of the DG [49]. We found that vMCs were inhibited in mice with fear generalization (Fig 5G), while activation of the vMC-dDG pathway produced biasedly activation of non-engram cells in the dDG, reducing the competitive advantage of engram cells in the dDG neural network and attenuating over-generalized fear (Figs 6F–6K and S8). Unlike previous studies that only emphasize the importance of the dDG in accurate memory retrieval [9,13,50], our results highlight the important role of interactions between dorsal and ventral regions within the DG in promoting accurate memory retrieval and preventing fear generalization.

On the other hand, when fear generalization is viewed as an efficient form of memory retrieval, the involvement of vMCs may be dispensable: following SS CFC training, mice exposed to all cues in the conditioned context do not require vMCs to regulate the activation ratio of mature engram cells within the dDG neuronal network.

### Modifying fear memory engram of dDG engram cells effectively rescues fear generalization

In addition, we confirm the presence of fear memory engrams within dDG neuronal ensembles activated during fear generalization. Pairing activation of these dDG neurons with exposure in an enriched environment results in a substantial reduction of fear generalization (Fig 7). These results imply that activated dDG neuronal ensemble during fear generalization mediates the occurrence and attenuation of fear generalization. Although mice were not directly exposed to an environment carrying natural cues, artificial activation of dDG neurons still induced the retrieval of fear memory, which initiated memory updating [51]. Long-term activation of these dDG engram cells results in cell-specific or synaptic-specific changes that destabilize the original memory [39,40], which may be responsible for attenuating fear generalization.

Fear generalization is difficult to attenuate, which makes PTSD patients difficult to cure [6,52]. Temporally modulating the activity of basolateral amygdala and prelimbic cortex has only real-time effects [45,53,54]. Unlike these regions, our research demonstrates the lasting effect of manipulating engram cells in the dDG to attenuate fear generalization, through switching the valence of contextual memory (Fig 7). While extinction learning reduces an individual’s fear expression in the short term, relapse frequently occurs in the long term as the extinction engrams only temporarily suppress the original fear engrams [55]. In contrast, modifying the original memory engram reverses its paired negative valence, leading to a permanent reduction in fear expression [39,56]. This phenomenon may also be perceived as a sustainable “extinction,” which could partly elucidate the mechanism behind exposure therapy for PTSD in clinical: it enables mice to recall fear memories, like patients, without re-entering the conditioned context.

Besides the vDG, the MS and the entorhinal cortex, 2 additional upstream brain regions of the dDG, might be involved in fear generalization as well. Previous studies have shown that excessive activity in the cholinergic MS circuitry promotes fear generalization [57]. In addition, lateral inhibition within the dDG enables interaction between different local neuronal ensembles, and may explain how different ensembles of dDG neurons, originating from the same upstream sources, exhibit varying activity during fear memory retrieval [25,58]. The mechanisms coordinating hippocampal activity during these memory processes merit further investigation beyond the scope of this study.

In summary, our study suggests that the activity of the dDG neuronal network, composed of both engram and non-engram cells, plays a critical role in accurate memory retrieval (Fig 8). In dDG, when engram cells associated with contextual fear memories dominate the activity of dDG neural network, mice exhibit generalized fear in a similar context. Conversely, reducing the competitive advantage of these cells or reversing their negative valence alleviates fear generalization. Notably, the activity of the vMCs-dDG pathway in the DG reduces the dominance of engram cells in the dDG neural network, thereby reducing fear generalization. Our findings provide insights into the dDG neuronal mechanism underlying fear generalization and offer potential strategies for alleviating fear generalization in patients with PTSD.

## Materials and methods

### Ethics statement

All experiments were approved by the Animal Care and Use Committee of Peking University Health Science Center (LA2020180).

### Animals

Male C57BL/6 mice were provided by the Department of Laboratory Animal Sciences, Peking University Health Science Center (Beijing, China). The cfos-tTA mice were acquired from Prof. Yanxue Xue (National Institute on Drug Dependence, Peking University, Beijing, China). The cfos-tTA mice were maintained on a diet containing 40 mg/kg doxycycline (Dox) (Readydietech, Shenzhen, China) for 2 weeks before receiving viral injection surgery. Dox-containing diet (Dox diet) was replaced with a standard Dox-free diet 60 h prior to behavioral tagging to open a time window of activity-dependent labeling. The cfos-tTA mice were put on a diet containing 100 mg/kg Dox immediately after desired behavioral labeling window to block any further neuronal tagging and were returned to the 40 mg/kg Dox diet the following day. Fos-CreER^T2^ mice were acquired from Prof. Peng Cao (Institute of Biophysics, Chinese Academy of Sciences). Ai9 mice were acquired from Prof. Haitao Wu (Beijing Institute of Basic Medical Sciences, Beijing, China). Fos-CreER^T2^ and Ai9 mice were crossed to generate Fos-CreER^T2^×Ai9 F1 mice. The Calb2-Cre mice were purchased from Shanghai Model Organisms Center, Inc. (Shanghai, China).

### Contextual fear conditioning (CFC)

Previous research suggests that after being exposed to high-intensity unconditioned stimuli, rodents exhibit increased fear responses to both conditioned stimuli and similar safe stimuli, demonstrating fear generalization [24, 59]. Specifically, in CFC training, administering more footshocks compared to receiving fewer footshocks leads to mice displaying high levels of fear response to both the original context and similar novel context during test [24]. In other words, the greater number of foot shocks induces fear generalization in mice. Therefore, we employed the following modeling approach:

Contextual fear conditioning training occurred in context A with unsignaled footshocks (2 s, 1.0 mA). Mice were individually placed into context A and allowed to explore for 3 min. Then, during the next 3 min, 3 footshocks or 6 footshocks were delivered. For the weak shocks experiment, contextual fear conditioning training contained 3 unsignaled footshocks, each separated by 58 s. Mice were removed from the apparatus 58 s after the last shock and returned to their homecage. For the strong shocks experiment, contextual fear conditioning training contained 6 unsignaled footshocks, each separated by 28 s. Mice were removed from the apparatus 28 s after the last shock and returned to their homecage. All mice were conditioned in context A.

### Fear generalization test and fear memory test

For the fear memory test, mice were tested in context A. Each test lasted 5 min.

For the fear generalization test, mice were tested in context B. Each test lasted 5 min.

### Stereotaxic injection

Mice were anesthetized with 1% sodium pentobarbitone (100 mg·kg^-1^) intraperitoneally (*i*.*p*.) and placed in a stereotactic head frame (RWD Life Science, Shenzhen, China) on a temperature-controlled heating pad (RWD Life Science, Shenzhen, China) to prevent hypothermia. Scalp hair was shaved and the skull skin was scrubbed with 70% alcohol. After exposing the skull via a midline incision, small holes were drilled through the skull at the appropriate coordinates for injection. Virus was injected using a glass micropipette connected to a 10-μl microsyringe (Gaoge, Shanghai, China) filled with mineral oil. The virus was bilaterally injected into dDG (0.3 μl/side; relative to bregma: AP, −1.8 mm; ML ± 1.2 mm; DV, −1.85 mm) or vDG (0.4 μl/side; relative to bregma: AP −3.7 mm; ML ± 2.7 mm; DV, −2.9 mm) at a flow rate of 0.1 μl/min via a microinjection pump (RWD Life Science, Shenzhen, China). After each injection, the needle was left in place for 5 min to allow for virus diffusion and then slowly withdrawn.

### Optogenetics

Following viral injection during the same surgery, 4 miniature stainless screws were fixed on the skull of mice used for optogenetic manipulation. The optic fiber was bilaterally implanted 0.2 mm dorsal to the virus injection site and was fixed on the skull with the screws and dental cement. The mouse remained on the heating pad until fully recovered from anesthesia. After surgery, mice were allowed to recover for 3 weeks before subsequent experiments. The light was bilaterally delivered from the laser to the mice via a fiber-optic patch cord, connected to a rotary joint that allowed mice to freely move.

For optogenetic activation, ChR2 was stimulated at 20 Hz in a square wave stimulation mode (7 mW, 3 s duration, 40% duty cycle) using a 473 nm laser (Hangzhou Newdoon Technology, Hangzhou, China) according to the following designated epochs. Testing sessions were 9 min, consisting of three 3-min epochs, with the first and third as light-off epochs, and the second as a light-on epoch. For optogenetic inhibition, a 560 nm laser (Hangzhou Newdoon Technology, Hangzhou, China) was used at 0.1 Hz in a square wave stimulation mode (7 mW, 3 s duration, 50% duty cycle) for the entire test session. In the end, the mouse was detached and returned to its homecage. The floors and walls of the test chamber were cleaned between mice.

### Chemogenetic manipulation

For chemogenetic manipulation of neuron activity, CNO-based excitatory DREADD (hM3Dq) or inhibitory DREADD (hM4Di) were expressed in desired neurons using the AAV virus. Clozapine N-oxide (CNO; Tocris, United Kingdom) was dissolved in normal saline. At 3 weeks after the microinjection of the virus, mice were injected CNO (1 mg/kg dissolved in ACSF) into dDG with the injection cannula 15 min or CNO (1 mg/kg dissolved in 0.9% saline) intraperitoneally (IP) 40 min prior to the behavioral test. Artificial cerebrospinal fluid (ACSF) containing NaCl (126 mM), KCl (2.5 mM), MgCl_2_ (1.3 mM), NaH_2_PO_4_ (1.2 mM), CaCl_2_ (2.4 mM), NaHCO_3_ (18 mM), and glucose (10 mM), pH 7.4, 290–300 mOsm.

For microinjection, we inject CNO bilaterally into dDG, 0.3 μl/side. The same amounts of ACSF were injected as controls. For intraperitoneal injecting, same amounts of normal saline were injected as controls.

### Immunostaining

Approximately 1.5 h after behavioral testing, mice were deeply anesthetized with 1% pentobarbital sodium (100 mg·kg^-1^, *i*.*p*.). Mice were transcardially perfused with 37°C normal saline, and then 4°C 4% paraformaldehyde (PFA) dissolved in 0.01 M phosphate buffer (PB, pH 7.4). The brains were removed and fixed in 4% PFA solution for 12 h, and subsequently dehydrated in 20% and 30% sucrose solutions in turn for >24 h at 4°C. Brains were embedded in optimal cutting temperature compound and sectioned coronally at 30-μm thickness using a freezing microtome (Model 3050s, Leica, Germany). Free-floating brain slices were washed with phosphate-buffered saline (PBS) 3 times for 5 min each and incubated in the blocking solution (PBS containing 0.3% Triton X-100 and 5% bull serum albumin) for 60 min at room temperature. Slices were then incubated with primary antibodies (1:200 dilution in blocking solution) at 4°C overnight. The following day, slices were washed 3 times with PBS for 10 min each and incubated in the secondary antibody (1:500 in blocking solution) for 1.5 h at room temperature. Following incubation with secondary antibody, brain sections were washed again 3 times with PBS for 10 min each. Finally, after washing, slices were mounted on microscope slides, and coverslips were mounted using a mounting medium containing DAPI (1:1,000). Images were captured using a laser-scanning confocal microscope (model FV1000, Olympus, Japan).

### Cell counting

Specific brain regions were annotated using Allen Brain Atlas as a reference (http://mouse.brain-map.org/static/atlas). The dDG and vDG were photographed and analyzed at −1.75 to −2.05 mm and −3.38 to −3.68 mm from bregma, respectively. Three slices per mouse were acquired and analyzed. The data per mouse was the average of the slices. All images belonging to the same experiment and displayed together were acquired with the same settings. All mCherry^+^ cells and DAPI-stained nuclei within designated brain regions were counted automatically using Fiji. The level of activation in different brain regions was normalized by calculating (mCherry^+^ neurons)/DAPI. A similar approach was applied for quantifying cFOS^+^ cells, cFOS^+^ mCherry^+^ overlap cells, Calb2^+^ cells, and overlap cells, etc.

### Pre-handling

Prior to CFC, all behavioral mice were individually habituated to handling by the investigator for 5 min on each of 3 separate days. Handling took place in the room where the mice were raised.

### Contexts

Context A was a chamber (25 × 22 × 29  cm) with black and white striped walls, steel grid floors, black square ceilings, and scented with 75% ethanol. Context A was cleaned with 75% ethanol between each run.

Context B was a chamber (25 × 22 × 29 cm) similar to context A. Context B consisted of gray grid walls and steel grid floors and was scented with 1% acetic acid. Context B was cleaned with 1% acetic acid between each run.

Context C was a cylindrical (21 cm in diameter and 30 cm in height) plastic container with white walls and flat Plexiglas floors, and had no detectable scent. Context C was cleaned using unscented disposable disinfecting wipes between each run.

The enriched environment cage was a large octahedral cage (60 × 40 × 32 cm) consisted of novel objects such as cubes in different shapes and colors, running wheels, platforms of different heights, and balls. Ad libitum food and water were provided in the enriched environment cage.

### Miniature two-photon calcium imaging recording and analysis

Mice were disposed of through similar procedures as the stereotaxic injection described above. After AAV2/9-hSyn-GCaMP6s virus injection into dDG, a 0.6-mm in diameter, approximately 4 mm in length gradient refractive index lens (GRIN lens; GoFoton, United States of America) was slowly advanced into dDG until the tip was placed at DV: −1.5 mm. After 3 to 4 weeks of viral expression, an imaging baseplate was positioned over the GRIN lens and cemented with denture base resins. The miniature two-photon microscope (FHIRMTPM V2.0, the field of view: 420 × 420 mm^2^; resolution: approximately 1.13 μm; working distance: 1 mm) was detachable while its holder was mounted permanently onto a baseplate over the GRIN lens. The cover of the holder and protective glue (Kwik-Cast, WPI Inc., USA) on the GRIN lens were removed before imaging. Then, headpiece was mounted on the holder and locked with M2 screws. Imaging data were acquired using imaging software (GINKGO-MTPM, Transcend Vivoscope Biotech Co., Ltd, China) at a frame rate of 10 Hz (512 × 512 pixels) with 920 nm and 1,030 nm femtosecond fiber laser (approximately 35 mW at the objective, TVS-FL-01, Transcend Vivoscope Biotech Co. Ltd, China). Timestamps of imaging frames were marked according to the controller (TVS-MMM-01, Transcend Vivoscope Biotech Co., Ltd, China).

To acclimate mice to the environment, each mouse was allowed to move freely with video recording. Each mouse was handled and adapted in the chamber. And each mouse was placed in homecage for 15 min per day in 3 consecutive days, before the first time of recording.

On day 0, mice were administered a WS or SS CFC paradigm in context A. On day 3, mice were placed to recording in the homecage for 5 min. Then, mice were administered the fear generalization test in context B. During homecage, and fear generalization test, calcium signals from dDG neurons in mice were recorded.

The homecage is an environment that is extremely familiar to mice. We did not observe freezing in mice in the homecage, which is different from the confounding caused by preexposure before contextual fear conditioning in previous studies [60].

Motion correction, registration, cell detection, and signal extraction were performed via Suite2p [61] followed by manual correction. Fluorescence value (F) traces were converted to a normalized trace using the following formula: ΔF/F_0_ = (F-F_0_)/F_0_. F_0_ was computed via a sliding-window of 500 frames (approximately 50 s) using a quartile cut-off that ranged from the bottom 10th percentile up to the median, depending on how active the neuron was [61,62]. Lastly, the ΔF/F traces were given by [F(i)-F_0_(i)]/F_0_(i), where i is the index for each frame. The final ΔF/F traces were standardized by subtracting the median and dividing by the standard deviation (SD).

Ca^2+^ transient event detection method refers to previous research reports [62,63]. To detect Ca^2+^ transient events, all fluorescence traces were deconvolved using the CASCADE [64] and were Z-scored with the mean calculated from time points lacking Ca^2+^ activity. Ca^2+^ transient events were then defined as transients exceeding a 3 SD amplitude from a 0.5 SD baseline, lasting a minimum duration (calculated by [-ln(A/A_0_)/t_half_], where A_0_ = 0.5 and A = amplitude of that transient; t_half_ for GCaMP6f was 200 ms, taken from before returning to a 0.5 SD baseline level. Additional Ca^2+^ transient rising events within detected Ca^2+^ transients that were large and multi-peaked were then detected using the findpeaks function in MATLAB (Mathworks, Natick, Massachusetts, USA) with the following parameters (MinPeakProminence = 2.5 SD, MinPeakDistance = 1 s).

### Cell selectivity analysis

We entirely preserved the temporal dynamics of the calcium activity trace and instead shuffled behavioral epochs to generate the null distribution. Epochs were thus fixed in length to account for bias in occupancy. In each of 10,000 iterations, the difference in average activity between the behavioral epoch (freezing state) and all other time points was calculated for comparison to the actual difference in activity. These shuffled differences form a null distribution. According to the actual difference, probability *p* values were calculated. Here, probability values of *p* < 0.01 were considered statistically significant. When *p* < 0.01, if the actual difference is greater than the shuffled difference, the neuron is defined as a Freezing neuron that responds specifically to freezing. If the actual difference is less than the scrambled difference, the neuron is defined as a Nonfreezing neuron that responds specifically to nonfreezing. When *p* ≥ 0.01, the neuron was defined as a nonselective neuron with no specific response.

### Experimental design of Fig 3

Experimental design of Fig 3A: The dDG of cfos-tTA mice were injected with AAV9-TRE3G-mCherry (WS-mCherry group) or AAV9-TRE3G-ChR2-mCherry (WS-ChR2 group) virus. The optic fiber was embedded above the dDG. Mice were fed with the Dox diet. After virus expression, Mice were taken off Dox for 2.5 days. Next, mice were subjected to CFC training under WS in context A. After CFC training, mice were put back on Dox. After 3 day, mice were placed in context B for fear generalization test. During the test, the laser was delivered, and 1.5 h after testing, mice were perfused for immunostaining.

Experimental design of Fig 3D: The dDG of cfos-tTA mice were injected with AAV9-TRE3G-mCherry (SS-mCherry group) or AAV9-TRE3G-ChR2-mCherry (WS-ChR2 group and SS-ChR2 group) virus. The optic fiber was embedded above the dDG. Mice were fed with the Dox diet. After virus expression, mice were subjected to CFC training under WS or SS in context A. Mice were taken off Dox for 2.5 days, tested in context B, and put back on Dox. After 1 day, mice were placed in context C for testing. During the test, the laser was delivered from the fourth minute to the sixth minute; 1.5 h after testing, mice were perfused for immunostaining.

Experimental design of Fig 3I: Top: After virus expression, mice were taken off Dox for 2.5 days, subjected to CFC training in context A, and put back on Dox. Three days later, mice were tested in context B. During the fear generalization test, the laser was delivered, and 1.5 h after testing, mice were perfused. Finally, immunostaining was performed. Bottom: Schematic diagram showing that EYFP cells in the NPHR group were optogenetically inhibited.

### Experimental design of Fig 4

Experimental design of Fig 4A: The dDG of cfos-tTA mice were injected with AAV9-TRE3G-mCherry (mCherry group) or AAV9-TRE3G-ChR2-mCherry (ChR2 group) virus. The optic fiber was embedded above the dDG. After virus expression, mice were taken off Dox for 2.5 days, placed in context C to label activated neurons, and put back on Dox. Three days later, mice were placed in context B to test, and 1.5 h after testing, mice were perfused for immunostaining.

Experimental design of Fig 4D: The dDG of cfos-tTA mice were injected with AAV9-TRE3G-mCherry (mCherry group) or AAV9-TRE3G-ChR2-mCherry (ChR2 group) virus. The optic fiber was embedded above the dDG. After virus expression, mice were taken off Dox for 2.5 days, placed in context C to label activated neurons, and put back on Dox. Three days later, mice were subjected to CFC training in context A. Three days later, mice were tested in context B with laser delivered.

Experimental design of Fig 4G: In EGFP group, the C57 mice were injected with AAV9-cfos-tta-TRE3G-mCherry and AAV9-hSyn-EGFP. In ChR2 group, the C57 mice were injected with AAV9-cfos-tta-TRE3G-mCherry and AAV9-hSyn-ChR2-EGFP. Optic fiber was embedded above the dDG. After virus expression, mice were taken off Dox for 2.5 days, subjected to CFC training in context A, and put back on Dox. Three days later, mice were tested in context B with laser delivered.

### Experiment design of enriched environment exploration

The experimental design of Fig 7A is as follows: The dDG of cfos-tTA mouse was injected with AAV9-TRE3G-mCherry or AAV9-TRE3G-hM3D(Gq)-mCherry virus. After the virus is fully expressed, mice were subjected to CFC training under SS in context A. For mCherry-Context B-CNO group and hM3D(Gq)- Context B-CNO group, mice were taken off Dox for 2.5 days and then tested in context B. Mice were put back on Dox. After 1 day, mice were intraperitoneally injected with CNO. And 40 min later, mice were placed in the enriched environment for 2 h. After the mice were put back into the homecage to rest for 2 h, they were put into the enriched environment again for 2 h. The next day, these operations were repeated. One day later, mice were placed in context B again for fear generalization test. For hM3D(Gq)-Homecage-CNO group, mice were put back on Dox before fear generalization test in context B 3 h. Therefore, only neurons activated in the homecage were labeled before the test in context B. For hM3D(Gq)-Homecage-Saline group, mice were injected with saline prior to the exploration of the enriched environment. Other operations are the same as mCherry-Context B-CNO group and hM3D(Gq)-Context B-CNO group.

### Quantification and statistical analysis

The Kolmogorov–Smirnov and Levene’s test was used to assess the normality of distribution and homogeneity for each variable. If the data conformed to the normal distribution and homogeneity of variance, data were expressed as mean ± SEM. Single variable comparisons were made with two-tail paired or unpaired Student’s *t* test. Group comparisons were made using either one-way or two-way analysis of variance (ANOVA) followed by Bonferroni post hoc tests. Data with no normal distribution and homogeneity were expressed as median ± range and nonparametric tests were used for variables. Specifically, for pair single variable comparisons, rank sum test was carried out in the comparisons of 2 groups. For unpair single variable comparisons, Mann–Whitney U test was used in the comparisons of 2 groups. For unpair single variable comparisons, Kruskal–Wallis test with Dunn’s post hoc test was used in the comparisons of 3 or more groups. A χ^2^ test was used to assess categorical data differences. For correlation analyses, Pearson correlation coefficients (Pearson r) and *p* values are reported. All statistics were calculated using GraphPad Prism or MATLAB. Probability values of *p* < 0.05 were considered statistically significant. Detailed statistical analysis was shown in S1 Data.

## Supporting information

S1 Fig Strong electric shock stimuli induce fear generalization. (A) Experimental design. To measure the baseline freezing levels of mice in both context A and context B, mice were placed in context A. After 3 days, mice were placed in context B. (B) The mice showed low levels (<10%) of freezing behavior in context A. (C) The mice showed low levels (<10%) of freezing behavior in context B. (D) Experimental design. Mice were subjected to CFC training under WS or SS in context A. After 3 days, mice were placed in context A for the fear memory test. (E) Both WS and SS groups exhibited high levels of freezing in context A during the fear memory test. (F) Experimental design. Mice were subjected to CFC training under WS or SS in context A. After 3 days, mice were placed in context B for the fear generalization test. (G) The SS group displayed a higher level of freezing during the fear generalization test in context B. In S1 Fig, statistical comparisons were performed using unpaired Student’s *t* test; data were presented as mean ± SEM. *p < 0.05, **p < 0.01. The underlying data and statistical information in S1 Fig can be found in S1 Data. The mice depicted were created with BioRender.com.
(TIF)

S2 Fig Labeled cells during CFC and activated cells during fear memory test and fear generalization test. (A) The proportion of engram cells (mCherry^+^) to the total cells (DAPI^+^). The proportion in the SS group was similar to that of the WS group. (B) The proportion of activated cells (cFOS^+^) to the total cells (DAPI^+^). The proportion in the SS group was similar to that of the WS group. (C) The proportion of activated engram cells (mCherry^+^ cFOS^+^) to the total cells (DAPI^+^). The proportion in the SS group was similar to that of the WS group. (D) The proportion of activated engram cells (mCherry^+^ cFOS^+^) to the total engram cells (mCherry^+^). The proportion in the SS group was similar to that of the WS group. (E) The proportion of engram cells (mCherry^+^) to the total cells (DAPI^+^). The proportion in the SS group was similar to that of the WS group. (F) The proportion of activated cells (cFOS^+^) to the total cells (DAPI^+^). Compared to the WS group, the SS group has fewer activated cells in dDG. (G) The proportion of activated engram cells (mCherry^+^ cFOS^+^) to the total cells (DAPI^+^). The proportion in the SS group was similar to that of the WS group. (H) The proportion of activated engram cells (mCherry^+^ cFOS^+^) to the total engram cells (mCherry^+^). The proportion in the SS group was similar to that of the WS group. In S2 Fig, statistical comparisons were performed using unpaired Student’s *t* test. Data were presented as mean ± SEM. *p < 0.05. The underlying data and statistical information in S2 Fig can be found in S1 Data.
(TIF)

S3 Fig Proportion of dDG neurons activated during homecage exploration. (A) and (B) Histograms of Ca^2+^ transients AUC/sec distribution for individual dDG neurons in the WS (A) and SS (B) groups during the exploration period in the homecage. (C) In the homecage, the proportions of inactive engram cells (inactive mCherry^+^) and non-engram cells (inactive mCherry^-^) among all inactive cells, respectively. The proportion was similar between WS and SS groups (Chi-squared test). (D) In the homecage, the proportions of active engram cells (active mCherry^+^) and non-engram cells (active mCherry^-^) among all active cells, respectively. The proportion was similar between WS and SS groups (Chi-squared test). The underlying data and statistical information in S3 Fig can be found in S1 Data.
(TIF)

S4 Fig Inhibition of dDG engram cells reduced the freezing levels in mice during fear memory test. (A) Virus injection. (B) Experimental design. Feed cfos-tTa mice with Dox diet. Then, inject AAV-TRE3G-EYFP (SS-EYFP group) or AAV-TRE3G-NpHR-EYFP (SS-NpHR group) virus into dDG in cfos-tTa mice. After the virus was fully expressed, mice were taken off Dox for 2.5 days and subjected to CFC training in context A, following by a diet with Dox. Three days later, mice were placed in context A for fear memory test. During the test, we applied laser stimulation to the dDG. After 1.5 h posttest completion, the mice were perfused, and their brains were harvested for immunostaining. (C) Representative images of EYFP^+^ expressing (green) and cFOS^+^ immunostaining (red) in the dDG. Nuclei in blue (DAPI). Scale bar, 100 μm. (D) Compared with SS-EYFP group, SS-NpHR group displayed a lower level of freezing during the fear memory test in context A. Statistical comparisons were performed using unpaired Student’s *t* test. Data were presented as mean ± SEM. *p < 0.05. The underlying data and statistical information in S4 Fig can be found in S1 Data. The mice depicted were created with BioRender.com.
(TIF)

S5 Fig Activated vMCs during fear memory test. (A) Experimental design. Left: After the virus was fully expressed, mice were taken off Dox for 2.5 days and subjected to CFC training in context A, following by a diet with Dox. Three days later, mice were placed in context A for fear memory test and perfused 1.5 h after for immunostaining. (B) Representative image of Calb2^+^ (red) and cFOS^+^ (green) immunostaining in the vDG. Nuclei were stained with DAPI (blue). Scale bar, 100 μm. (C) The proportion of vMCs (Calb2^+^) to all cells (DAPI^+^). Similar percentage of vMCs among all cells in the vDG hilus between the WS and SS groups. (D) The proportion of activated vMCs (Calb2^+^ cFOS^+^) to all cells (DAPI^+^). Similar percentage of activated vMCs among all cells between the WS and SS groups. (E) The proportion of activated vMCs (Calb2^+^ cFOS^+^) to all vMCs (Calb2^+^). Similar percentage of activated vMCs among vMCs in the vDG hilus between the WS and SS groups. In S5C–S5E Fig, statistical comparisons were performed using unpaired *t* tests. Data were presented as mean ± SEM. The underlying data and statistical information in S5 Fig can be found in S1 Data. The mice depicted were created with BioRender.com.
(TIF)

S6 Fig Inhibiting vMCs does not induce fear generalization. (A) Virus injection: The vDG of Calb2-Cre mice were injected with AAV-hSyn-DIO-hM4D(Gi)-mCherry virus. (B) Experimental design: After virus expression, mice were subjected to CFC training under WS in context A. After 3 days, mice were intraperitoneally injected with saline (Saline group) or CNO (CNO group); 40 min later, mice were placed in context B for the fear generalization test, and 1.5 h after testing, mice were perfused for immunostaining. (C) Representative images of mCherry^+^ expression (red) and cFOS^+^ immunostaining (green) in the dDG (top) and vDG (bottom). Left: Saline group. Right: CNO group. Nuclei were stained with DAPI (blue). Scale bar, 100 μm. (D) The Saline and CNO groups showed a similar freezing level in context B during the fear generalization test. (E) The percentage of cFOS^+^ cells. The dDG granular cell layer of the CNO group displayed a significantly lower percentage of activated dDG cells (cFOS^+^) than that of the saline group. (F) Experimental design. Left: The vDG of Calb2-Cre mice were injected with AAV-hSyn-DIO-mCherry virus. Right: After virus expression, mice were subjected to CFC training under WS in context A. After 3 days, mice were intraperitoneally injected with saline (Saline group) or CNO (CNO group), and 40 min later, mice were placed in context B for the fear generalization test, and 1.5 h after testing, mice were perfused for immunostaining. (G) Representative images of mCherry^+^ expression (red) and cFOS^+^ immunostaining (green) in the dDG (top) and vDG (bottom). Left: Saline group. Right: CNO group. Nuclei were stained with DAPI (blue). Scale bar, 100 μm. (H) The Saline and CNO groups showed a similar freezing level in context B during the fear generalization test. (I) The percentage of cFOS^+^ cells. The dDG granular cell layer of the CNO group showed a similar percentage of activated dDG cells (cFOS^+^) with that of the saline group. Statistical comparisons were performed using unpaired Student’s *t* test. Data were presented as mean ± SEM. The underlying data and statistical information in S6 Fig can be found in S1 Data. The mice depicted were created with BioRender.com.
(TIF)

S7 Fig Injection of CNO alone does not affect dDG activity or fear generalization. (A) Experimental design. Left: The vDG of Calb2-Cre mice were injected with AAV-hSyn-DIO-EGFP virus. Right: After virus expression, mice were subjected to CFC training under SS in context A. After 3 days, mice were intraperitoneally injected with saline (Saline group) or CNO (CNO group), and 40 min later, mice were placed in context A (fear memory test) or context B (fear generalization test), and 1.5 h after testing, mice were perfused for immunostaining. (B) Representative images of EGFP^+^ expressing (green) and cFOS^+^ immunostaining (red) in the dDG (top) and vDG (bottom). Left: Saline group. Right: CNO group. EGFP^+^ fibers indicated axons in the dDG originating from the vMCs cells. Nuclei in blue (DAPI). Scale bar, 100 μm. (C) The freezing level of mice during the fear memory test. The Saline and CNO groups showed a similar freezing level in context A during the fear memory test. (D) The freezing level of mice during the fear generalization test. The Saline and CNO groups showed a similar freezing level during the fear generalization test in context B. (E) The percentage of cFOS^+^ cells. The dDG granular cells of CNO group and Saline group displayed a similar percentage of activated dDG cells (cFOS^+^). (F and G) Experimental design. (F) The vDG of Calb2-Cre mice was injected with AAV-hSyn-DIO-EGFP virus and the dDG were injected with AAV-cfos-tTA-TRE3G-mCherry virus. (G) After virus expression, mice were subjected to CFC training under SS in context A. After 3 days, mice were injected with ACSF (ACSF group) or CNO (CNO group) intracranially with the injection cannula; 15 min later, mice were placed in context B to test, and 1.5 h after testing, mice were perfused for immunostaining. (H) Representative images of EGFP^+^ expression (white), mCherry^+^ expression (red), and cFOS^+^ immunostaining (green) in the dDG. Left: Saline group. Right: CNO group. Nuclei were stained with DAPI (blue). Scale bar, 100 μm. (I) The CNO group and ACSF group displayed a similar level of freezing during the fear generalization test in context B. (J) The percentage of cFOS^+^ cells. The dDG granular cells of CNO group displayed a similar percentage of activated dDG cells (cFOS^+^) with that of the ACSF group. (K) Activated engram cells (mCherry^+^ cFOS^+^)/activated cells (cFOS^+^). The proportion of activated engram cells among all activated cells in the CNO group is similar with ACSF group. Statistical comparisons were performed using unpaired Student’s *t* test. Data were presented as mean ± SEM. The underlying data and statistical information in S7 Fig can be found in S1 Data. The mice depicted were created with BioRender.com.
(TIF)

S8 Fig Labeled cells and activated cells in dDG. (A) The proportion of engram cells (mCherry^+^) to the total cells (DAPI^+^). The proportion in the CNO group was similar to that of the ACSF group. (B) The proportion of activated engram cells (mCherry^+^ cFOS^+^) to the total cells (DAPI^+^). The proportion in the CNO group was similar to that of the ACSF group. (C) The proportion of activated engram cells (mCherry^+^ cFOS^+^) to the total engram cells (mCherry^+^). The proportion in the CNO group was similar to that of the ACSF group. (D) Activated engram cells (mCherry^+^ cFOS^+^)/engram cells (mCherry^+^). The proportion of activated engram cells in the CNO group was similar to that of the ACSF group. (E) Activated non-engram cells (mCherry^-^ cFOS^+^)/non-engram cells (mCherry^-^). The proportion of non-engram cells activated in the CNO group was significantly higher than in the ACSF group. Statistical comparisons were performed using unpaired Student’s *t* test. Data were presented as mean ± SEM. *p < 0.05. The underlying data and statistical information in S8 Fig can be found in S1 Data.
(TIF)

S1 Data Summary of statistical analysis. (XLSX)

## Abbreviations

ACSF: artificial cerebrospinal fluid
BLA: basolateral amygdala
CFC: contextual fear conditioning
dDG: dorsal dentate gyrus
MnR: median raphe
MS: medial septum
PBS: phosphate-buffered saline
PFA: paraformaldehyde
PTSD: posttraumatic stress disorder
SD: standard deviation
SS: strong shock
vDG: ventral dentate gyrus
WS: weak shock
