# Supplementary figures and images for: Dominant activities of fear engram cells in the dorsal dentate gyrus underlie fear generalization in mice
Source: PLoS Biol. 2024 Jul 12;22(7):e3002679. doi: 10.1371/journal.pbio.3002679 (PMC11244812; doi:10.1371/journal.pbio.3002679)

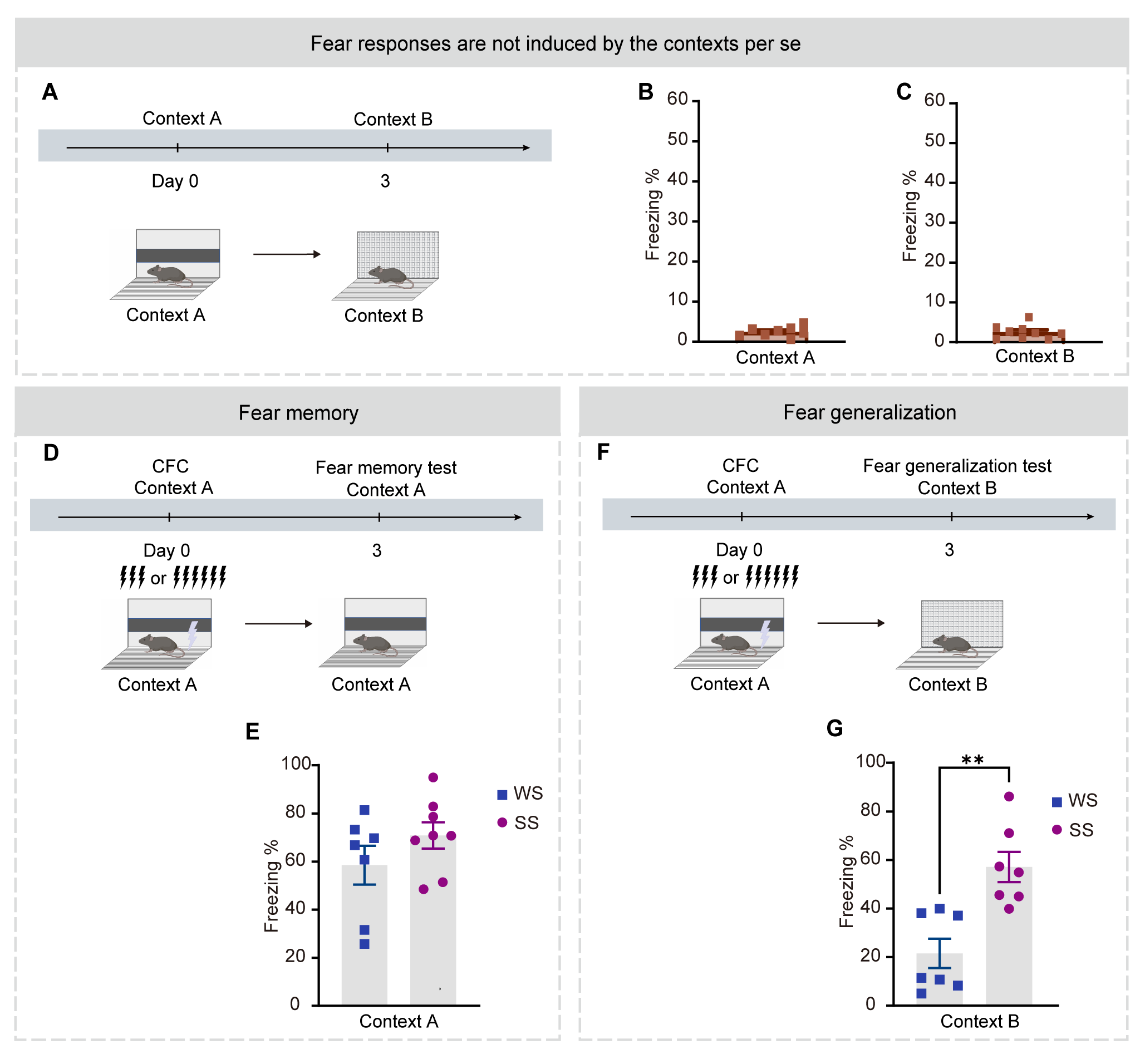

Supplement: S1 Fig — (A) Experimental design. To measure the baseline freezing levels of mice in both context A and context B, mice were placed in context A. After 3 days, mice were placed in context B. (B) The mice showed low levels (<10%) of freezing behavior in context A. (C) The mice showed low levels (<10%) of freezing behavior in context B. (D) Experimental design. Mice were subjected to CFC training under WS or SS in context A. After 3 days, mice were placed in context A for the fear memory test. (E) Both WS and SS groups exhibited high levels of freezing in context A during the fear memory test. (F) Experimental design. Mice were subjected to CFC training under WS or SS in context A. After 3 days, mice were placed in context B for the fear generalization test. (G) The SS group displayed a higher level of freezing during the fear generalization test in context B. In S1 Fig, statistical comparisons were performed using unpaired Student’s t test; data were presented as mean ± SEM. *p < 0.05, **p < 0.01. The underlying data and statistical information in S1 Fig can be found in S1 Data. The mice depicted were created with BioRender.com. (TIF) [file pbio.3002679.s001.tif]

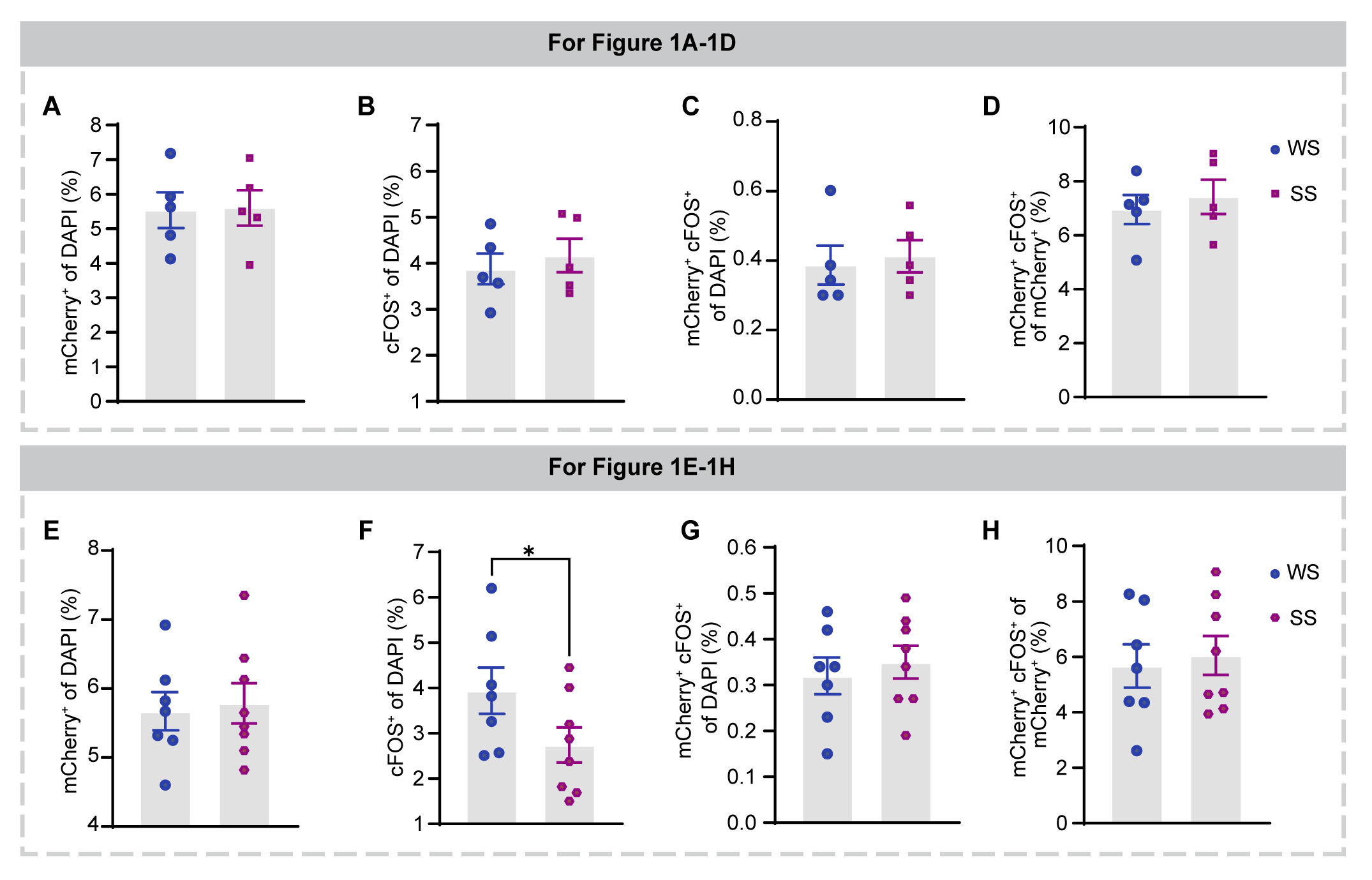

Supplement: S2 Fig — (A) The proportion of engram cells (mCherry+) to the total cells (DAPI+). The proportion in the SS group was similar to that of the WS group. (B) The proportion of activated cells (cFOS+) to the total cells (DAPI+). The proportion in the SS group was similar to that of the WS group. (C) The proportion of activated engram cells (mCherry+ cFOS+) to the total cells (DAPI+). The proportion in the SS group was similar to that of the WS group. (D) The proportion of activated engram cells (mCherry+ cFOS+) to the total engram cells (mCherry+). The proportion in the SS group was similar to that of the WS group. (E) The proportion of engram cells (mCherry+) to the total cells (DAPI+). The proportion in the SS group was similar to that of the WS group. (F) The proportion of activated cells (cFOS+) to the total cells (DAPI+). Compared to the WS group, the SS group has fewer activated cells in dDG. (G) The proportion of activated engram cells (mCherry+ cFOS+) to the total cells (DAPI+). The proportion in the SS group was similar to that of the WS group. (H) The proportion of activated engram cells (mCherry+ cFOS+) to the total engram cells (mCherry+). The proportion in the SS group was similar to that of the WS group. In S2 Fig, statistical comparisons were performed using unpaired Student’s t test. Data were presented as mean ± SEM. *p < 0.05. The underlying data and statistical information in S2 Fig can be found in S1 Data. (TIF) [file pbio.3002679.s002.tif]

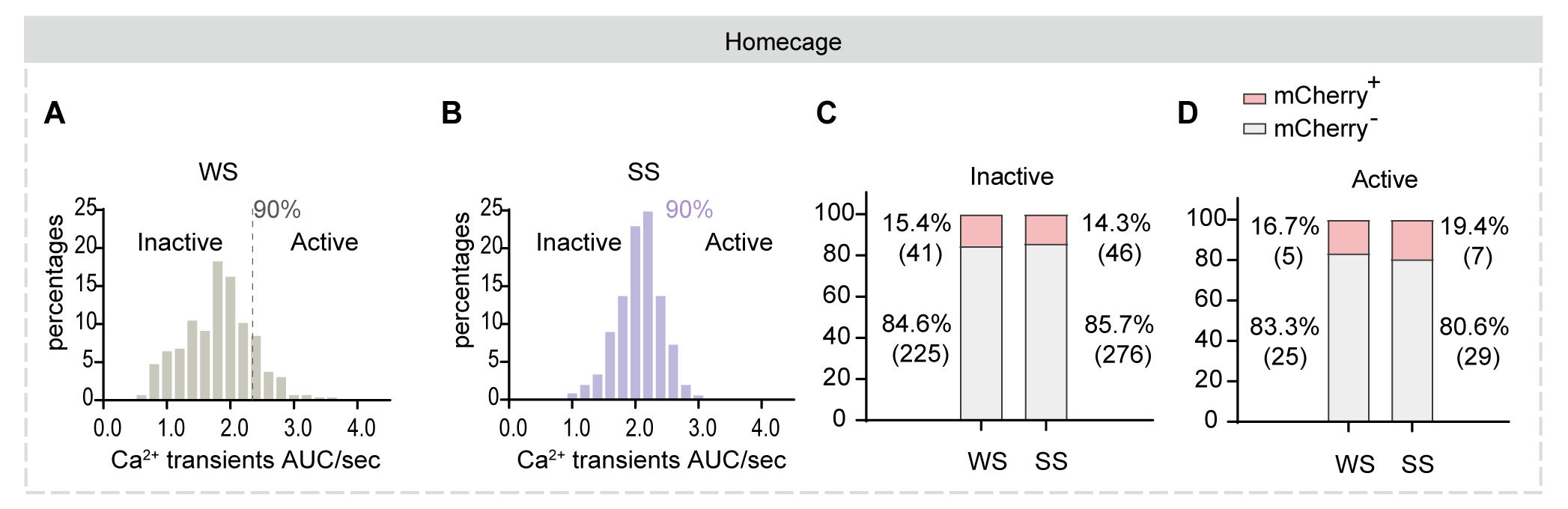

Supplement: S3 Fig — (A) and (B) Histograms of Ca2+ transients AUC/sec distribution for individual dDG neurons in the WS (A) and SS (B) groups during the exploration period in the homecage. (C) In the homecage, the proportions of inactive engram cells (inactive mCherry+) and non-engram cells (inactive mCherry-) among all inactive cells, respectively. The proportion was similar between WS and SS groups (Chi-squared test). (D) In the homecage, the proportions of active engram cells (active mCherry+) and non-engram cells (active mCherry-) among all active cells, respectively. The proportion was similar between WS and SS groups (Chi-squared test). The underlying data and statistical information in S3 Fig can be found in S1 Data. (TIF) [file pbio.3002679.s003.tif]

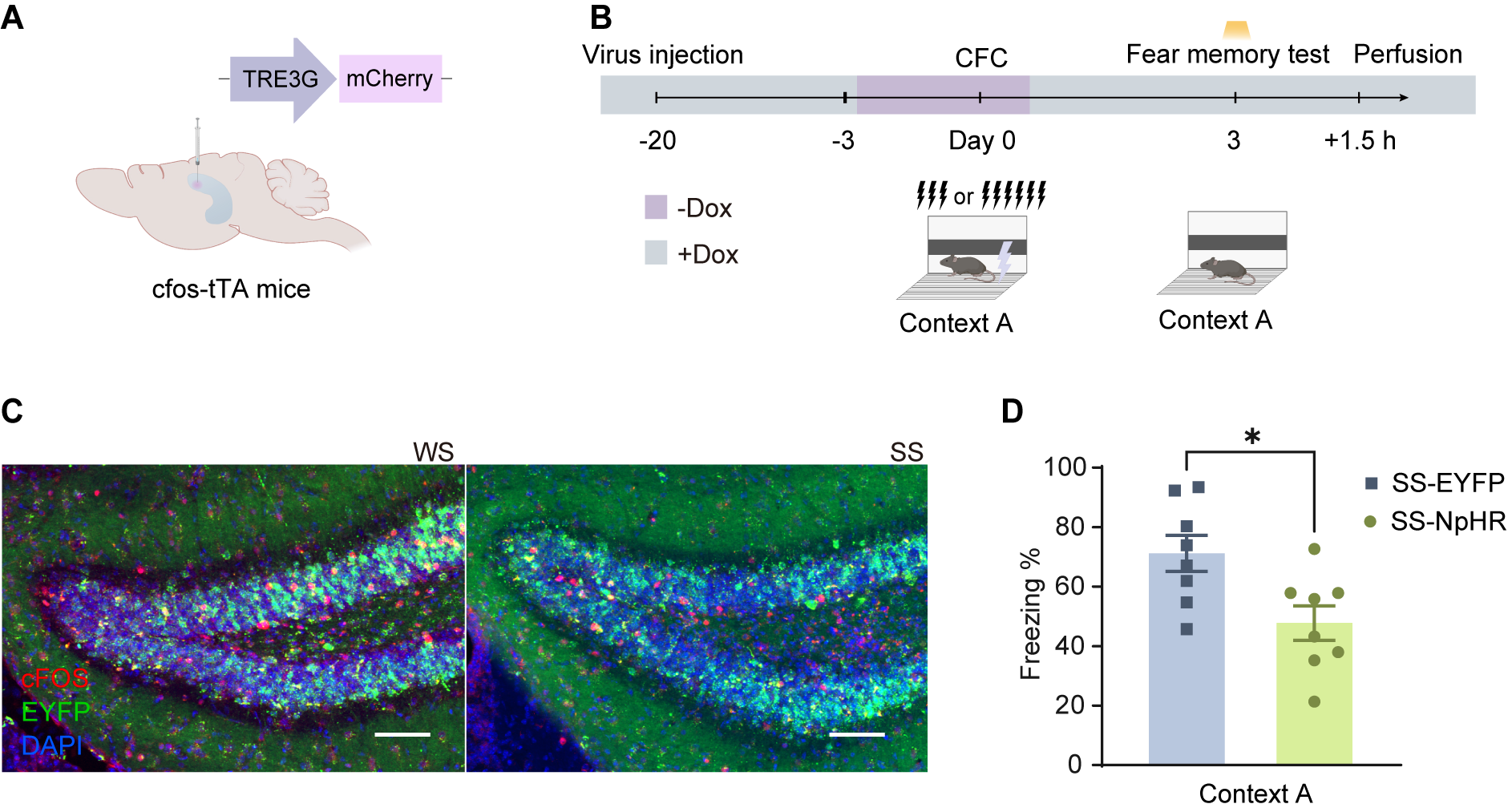

Supplement: S4 Fig — (A) Virus injection. (B) Experimental design. Feed cfos-tTa mice with Dox diet. Then, inject AAV-TRE3G-EYFP (SS-EYFP group) or AAV-TRE3G-NpHR-EYFP (SS-NpHR group) virus into dDG in cfos-tTa mice. After the virus was fully expressed, mice were taken off Dox for 2.5 days and subjected to CFC training in context A, following by a diet with Dox. Three days later, mice were placed in context A for fear memory test. During the test, we applied laser stimulation to the dDG. After 1.5 h posttest completion, the mice were perfused, and their brains were harvested for immunostaining. (C) Representative images of EYFP+ expressing (green) and cFOS+ immunostaining (red) in the dDG. Nuclei in blue (DAPI). Scale bar, 100 μm. (D) Compared with SS-EYFP group, SS-NpHR group displayed a lower level of freezing during the fear memory test in context A. Statistical comparisons were performed using unpaired Student’s t test. Data were presented as mean ± SEM. *p < 0.05. The underlying data and statistical information in S4 Fig can be found in S1 Data. The mice depicted were created with BioRender.com. (TIF) [file pbio.3002679.s004.tif]

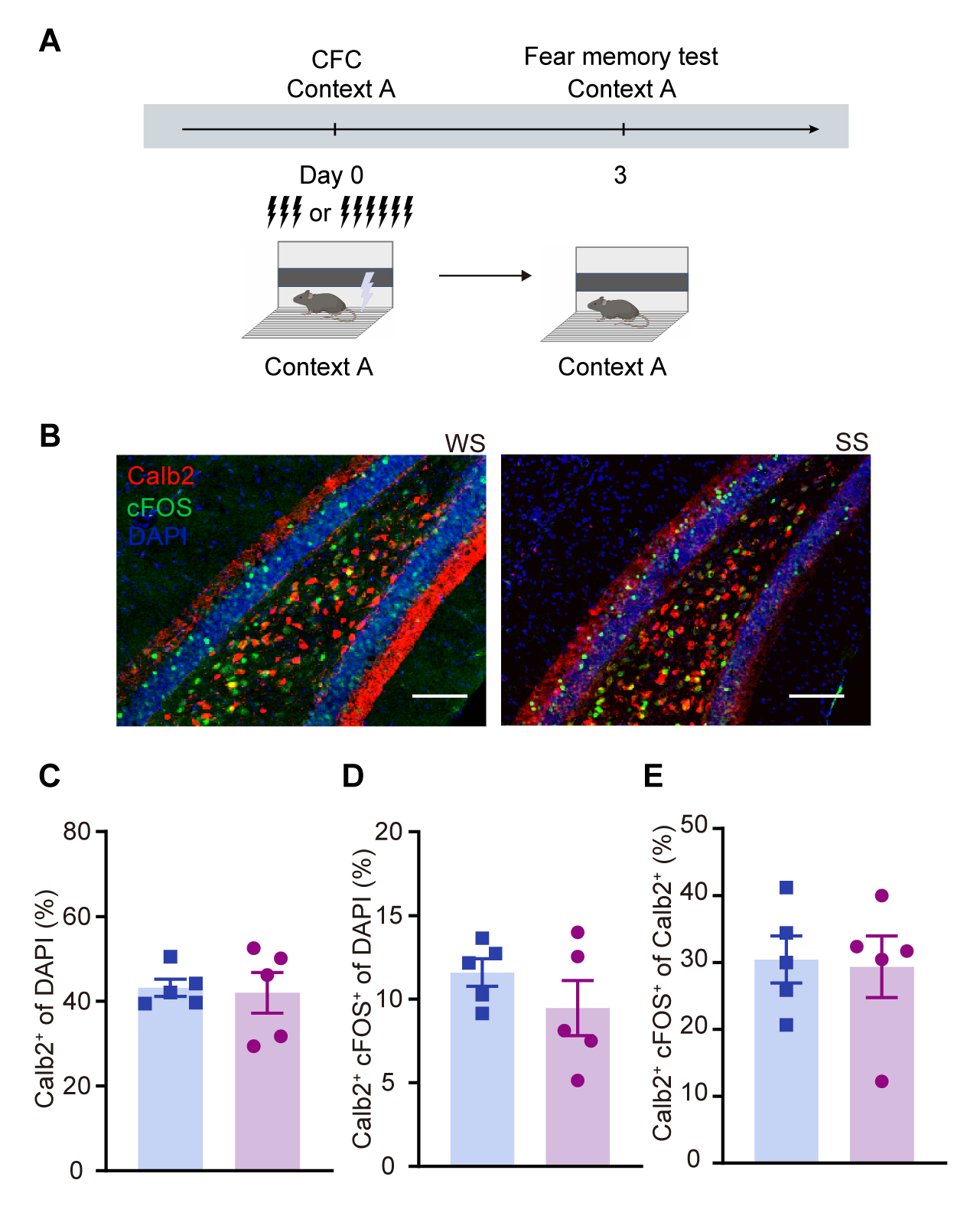

Supplement: S5 Fig — (A) Experimental design. Left: After the virus was fully expressed, mice were taken off Dox for 2.5 days and subjected to CFC training in context A, following by a diet with Dox. Three days later, mice were placed in context A for fear memory test and perfused 1.5 h after for immunostaining. (B) Representative image of Calb2+ (red) and cFOS+ (green) immunostaining in the vDG. Nuclei were stained with DAPI (blue). Scale bar, 100 μm. (C) The proportion of vMCs (Calb2+) to all cells (DAPI+). Similar percentage of vMCs among all cells in the vDG hilus between the WS and SS groups. (D) The proportion of activated vMCs (Calb2+ cFOS+) to all cells (DAPI+). Similar percentage of activated vMCs among all cells between the WS and SS groups. (E) The proportion of activated vMCs (Calb2+ cFOS+) to all vMCs (Calb2+). Similar percentage of activated vMCs among vMCs in the vDG hilus between the WS and SS groups. In S5C–S5E Fig, statistical comparisons were performed using unpaired t tests. Data were presented as mean ± SEM. The underlying data and statistical information in S5 Fig can be found in S1 Data. The mice depicted were created with BioRender.com. (TIF) [file pbio.3002679.s005.tif]

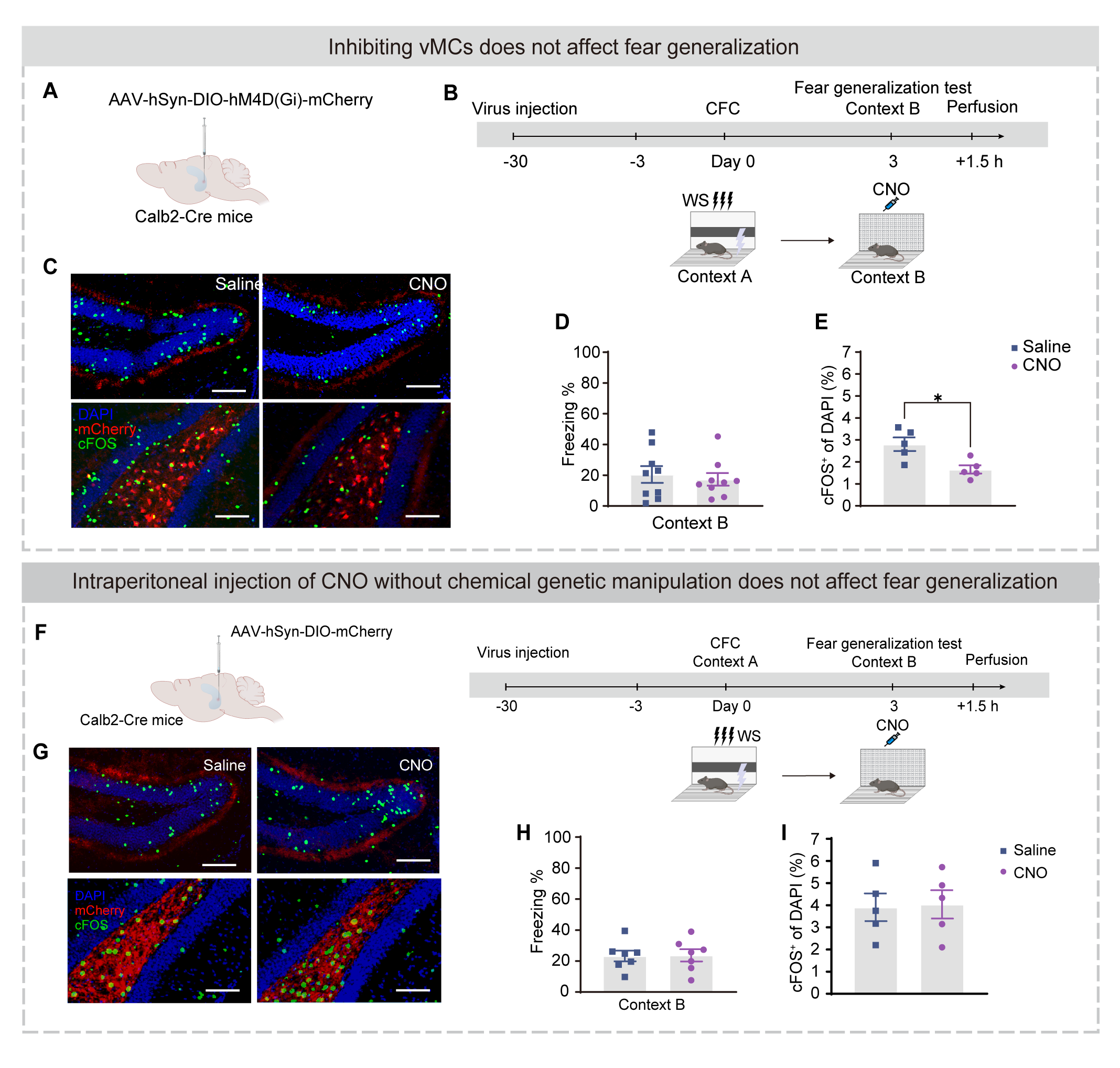

Supplement: S6 Fig — (A) Virus injection: The vDG of Calb2-Cre mice were injected with AAV-hSyn-DIO-hM4D(Gi)-mCherry virus. (B) Experimental design: After virus expression, mice were subjected to CFC training under WS in context A. After 3 days, mice were intraperitoneally injected with saline (Saline group) or CNO (CNO group); 40 min later, mice were placed in context B for the fear generalization test, and 1.5 h after testing, mice were perfused for immunostaining. (C) Representative images of mCherry+ expression (red) and cFOS+ immunostaining (green) in the dDG (top) and vDG (bottom). Left: Saline group. Right: CNO group. Nuclei were stained with DAPI (blue). Scale bar, 100 μm. (D) The Saline and CNO groups showed a similar freezing level in context B during the fear generalization test. (E) The percentage of cFOS+ cells. The dDG granular cell layer of the CNO group displayed a significantly lower percentage of activated dDG cells (cFOS+) than that of the saline group. (F) Experimental design. Left: The vDG of Calb2-Cre mice were injected with AAV-hSyn-DIO-mCherry virus. Right: After virus expression, mice were subjected to CFC training under WS in context A. After 3 days, mice were intraperitoneally injected with saline (Saline group) or CNO (CNO group), and 40 min later, mice were placed in context B for the fear generalization test, and 1.5 h after testing, mice were perfused for immunostaining. (G) Representative images of mCherry+ expression (red) and cFOS+ immunostaining (green) in the dDG (top) and vDG (bottom). Left: Saline group. Right: CNO group. Nuclei were stained with DAPI (blue). Scale bar, 100 μm. (H) The Saline and CNO groups showed a similar freezing level in context B during the fear generalization test. (I) The percentage of cFOS+ cells. The dDG granular cell layer of the CNO group showed a similar percentage of activated dDG cells (cFOS+) with that of the saline group. Statistical comparisons were performed using unpaired Student’s t test. Data were presented as m [file pbio.3002679.s006.tif]

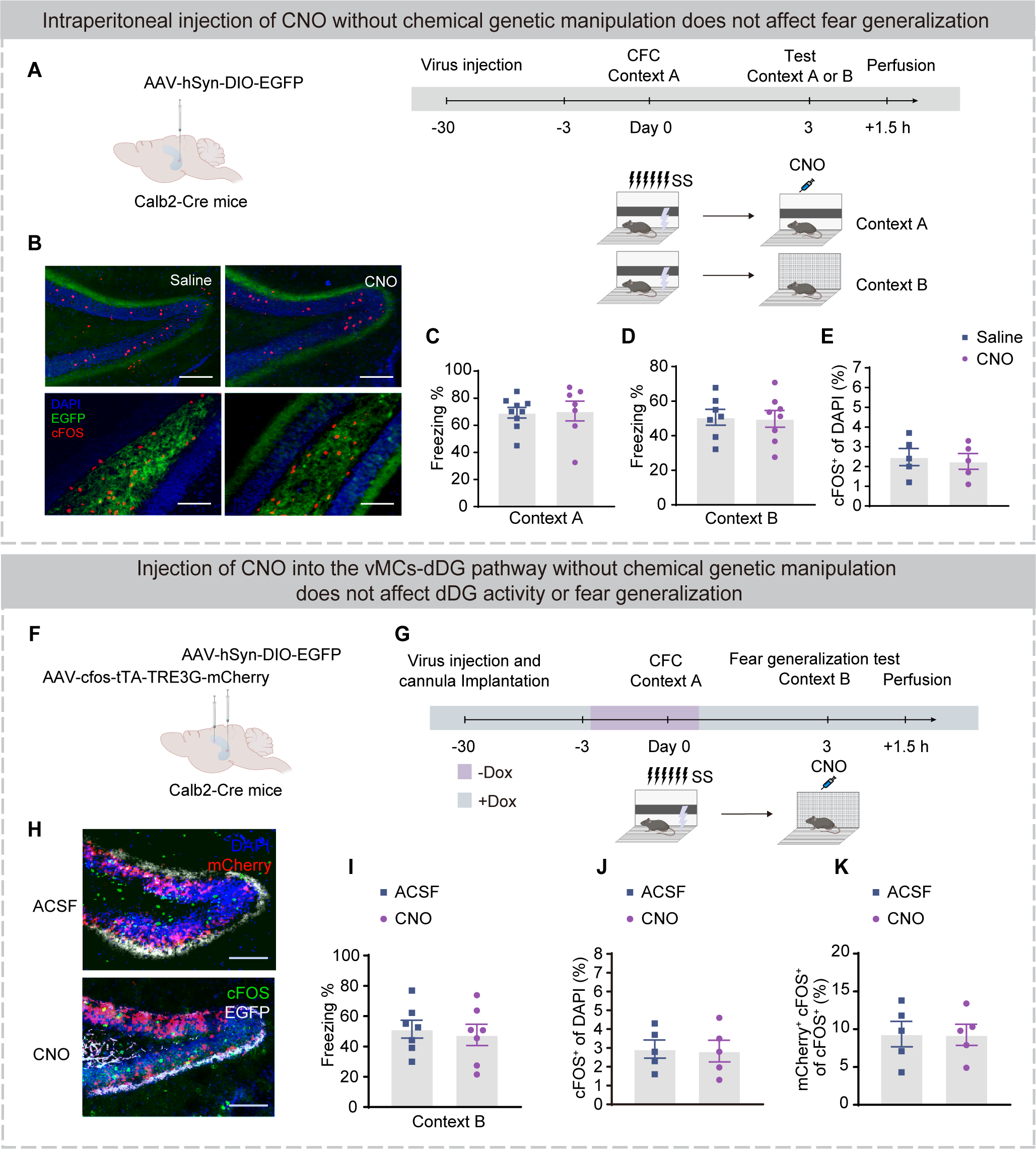

Supplement: S7 Fig — (A) Experimental design. Left: The vDG of Calb2-Cre mice were injected with AAV-hSyn-DIO-EGFP virus. Right: After virus expression, mice were subjected to CFC training under SS in context A. After 3 days, mice were intraperitoneally injected with saline (Saline group) or CNO (CNO group), and 40 min later, mice were placed in context A (fear memory test) or context B (fear generalization test), and 1.5 h after testing, mice were perfused for immunostaining. (B) Representative images of EGFP+ expressing (green) and cFOS+ immunostaining (red) in the dDG (top) and vDG (bottom). Left: Saline group. Right: CNO group. EGFP+ fibers indicated axons in the dDG originating from the vMCs cells. Nuclei in blue (DAPI). Scale bar, 100 μm. (C) The freezing level of mice during the fear memory test. The Saline and CNO groups showed a similar freezing level in context A during the fear memory test. (D) The freezing level of mice during the fear generalization test. The Saline and CNO groups showed a similar freezing level during the fear generalization test in context B. (E) The percentage of cFOS+ cells. The dDG granular cells of CNO group and Saline group displayed a similar percentage of activated dDG cells (cFOS+). (F and G) Experimental design. (F) The vDG of Calb2-Cre mice was injected with AAV-hSyn-DIO-EGFP virus and the dDG were injected with AAV-cfos-tTA-TRE3G-mCherry virus. (G) After virus expression, mice were subjected to CFC training under SS in context A. After 3 days, mice were injected with ACSF (ACSF group) or CNO (CNO group) intracranially with the injection cannula; 15 min later, mice were placed in context B to test, and 1.5 h after testing, mice were perfused for immunostaining. (H) Representative images of EGFP+ expression (white), mCherry+ expression (red), and cFOS+ immunostaining (green) in the dDG. Left: Saline group. Right: CNO group. Nuclei were stained with DAPI (blue). Scale bar, 100 μm. (I) The CNO group and ACSF group displayed a similar level of freez [file pbio.3002679.s007.tif]

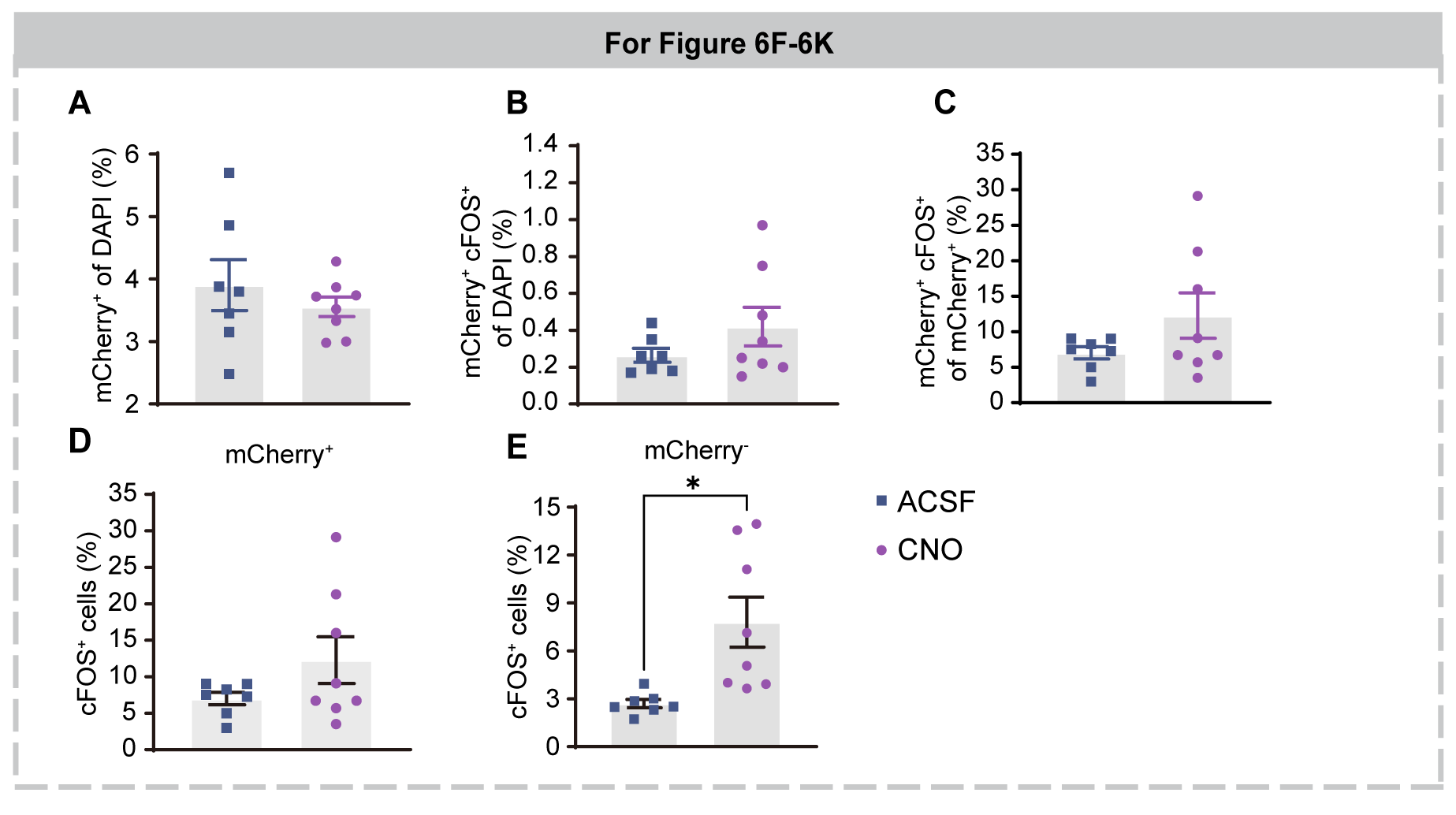

Supplement: S8 Fig — (A) The proportion of engram cells (mCherry+) to the total cells (DAPI+). The proportion in the CNO group was similar to that of the ACSF group. (B) The proportion of activated engram cells (mCherry+ cFOS+) to the total cells (DAPI+). The proportion in the CNO group was similar to that of the ACSF group. (C) The proportion of activated engram cells (mCherry+ cFOS+) to the total engram cells (mCherry+). The proportion in the CNO group was similar to that of the ACSF group. (D) Activated engram cells (mCherry+ cFOS+)/engram cells (mCherry+). The proportion of activated engram cells in the CNO group was similar to that of the ACSF group. (E) Activated non-engram cells (mCherry- cFOS+)/non-engram cells (mCherry-). The proportion of non-engram cells activated in the CNO group was significantly higher than in the ACSF group. Statistical comparisons were performed using unpaired Student’s t test. Data were presented as mean ± SEM. *p < 0.05. The underlying data and statistical information in S8 Fig can be found in S1 Data. (TIF) [file pbio.3002679.s008.tif]
